# *Staphylococcus aureus* PhoU Homologs Regulate Persister Formation and Virulence

**DOI:** 10.3389/fmicb.2020.00865

**Published:** 2020-05-26

**Authors:** Yongpeng Shang, Xiaofei Wang, Zhong Chen, Zhihui Lyu, Zhiwei Lin, Jinxin Zheng, Yang Wu, Qiwen Deng, Zhijian Yu, Ying Zhang, Di Qu

**Affiliations:** ^1^Key Laboratory of Medical Molecular Virology of MOE and MOH, Department of Medical Microbiology and Parasitology, School of Basic Medical Sciences, Fudan University, Shanghai, China; ^2^Department of Infectious Diseases and Shenzhen Key Lab for Endogenous Infection, Union Shenzhen Hospital, Huazhong University of Science and Technology, Shenzhen, China; ^3^Department of Molecular Microbiology and Immunology, Bloomberg School of Public Health, Johns Hopkins University, Baltimore, MD, United States

**Keywords:** persisters, PhoU homolog, *Staphylococcus aureus*, virulence, phosphate metabolism, ATP

## Abstract

PhoU homologs are one of the determinant factors in the regulation of persister formation and phosphate metabolism in many bacterial species; however, the functions of PhoU homologs exhibit species-specific characteristics. The pathogenesis of *Staphylococcus aureus* is closely correlated with persister formation and virulence factors. The functions of two PhoU homologs, PhoU1 and PhoU2, in *S. aureus* are unclear yet. In this study, single- and double-deletion mutants of *phoU1* and *phoU2* were generated in strain USA500 2395. The Δ*phoU1* or Δ*phoU2* mutants displayed a change in persister formation and virulence compared to the parent strain; the persisters to vancomycin and levofloxacin were decreased at least 1,000-fold, and the number of intracellular bacteria surviving in the A549 cells for 24 h decreased to 82 or 85%. The α-hemolysin expression and activity were increased in the Δ*phoU2* mutants. Transcriptome analysis revealed that 573 or 285 genes were differentially expressed by at least 2.0-fold in the Δ*phoU1* or Δ*phoU2* mutant vs. the wild type. Genes involved in carbon and pyruvate metabolism were up-regulated, and virulence genes and virulence regulatory genes were down-regulated, including type VII secretion system, serine protease, leukocidin, global regulator (*sarA*, *rot*), and the two-component signal transduction system (*saeS*). Correspondingly, the deletion of the *phoU1* or *phoU2* resulted in increased levels of intracellular pyruvate and ATP. Deletion of the *phoU2*, but not the *phoU1*, resulted in the up-regulation of inorganic phosphate transport genes and increased levels of intracellular inorganic polyphosphate. In conclusion, both PhoU1 and PhoU2 in *S. aureus* regulate virulence by the down-regulation of multiple virulence factors (type VII secretion system, serine protease, and leucocidin) and the persister generation by hyperactive carbon metabolism accompanied by increasing intracellular ATP. The results in *S. aureus* are different from what we have previously found in *Staphylococcus epidermis*, where only PhoU2 regulates biofilm and persister formation. The different functions of PhoU homologs between the two species of *Staphylococcus* warrant further investigation.

## Introduction

*Staphylococcus aureus* is a human pathogen that colonizes human skin and mucous membranes (Otto, 2010; Tong et al., 2015). It can invade phagocytic, epithelial, or endothelial cells and allows for the formation of persisters that can cause chronic and recurrent infections (Conlon, 2014). Moreover, the pathogenicity of *S. aureus* is closely correlated to its virulence factors (such as hemolysins, leukotoxins, enterotoxin, and coagulase) and biofilm formation (Dinges et al., 2000; Otto, 2014). Persisters are a subpopulation of bacterial cells that are tolerant to antibiotics without changes in minimum inhibitory concentration (MIC) values in the whole population and are one of the most important factors in the failure of antibiotic therapy (Wilmaerts et al., 2019). Persister formation is often explained by multiple mechanisms such as the reduction of cellular energy, cessation of DNA replication, blocked transcription and translation, decreased intracellular antibiotic concentrations, and antibiotic-induced damage (El-Halfawy and Valvano, 2015; Fisher et al., 2017; Wilmaerts et al., 2019). PhoU homologs are associated with persister formation in species including *Escherichia coli*, *Pseudomonas aeruginosa*, *Mycobacterium tuberculosis*, and *Staphylococcus epidermidis* (Li and Zhang, 2007; Shi and Zhang, 2010; Wang et al., 2017). The biological functions of the PhoU homologs in *S. aureus* are unclear and still require further investigation.

PhoU orthologs are found in many species of bacteria, but not in humans, and have been identified as phosphate-specific transport system accessory proteins (Morohoshi et al., 2002; Wang et al., 2013; de Almeida et al., 2015). In *E. coli*, PhoU protein is involved in the response to environmental Pi levels by interacting with PhoR and PstB (Gardner et al., 2014). A crystal structure analysis of PhoU protein in *Thermotoga maritima* revealed multinuclear iron clusters by a conserved E(D)XXXD motif pair (Liu et al., 2005). Little is known about the function of PhoU besides being a phosphate regulator. One *phoU* homolog is in the *pst* operon of *E. coli* and *P. aeruginosa*, whereas two *phoU* homologs are found in *M. tuberculosis*, *Mycobacterium marinum*, *S. epidermidis*, and *S. aureus*. In 2007, Li and Zhang (2007) showed that the inactivation of *phoU* in *E. coli* resulted in persister reduction and an up-regulated transcription level of some functional genes involved in energy production, nutrient transportation, flagellar synthesis, and chemotaxis. In *P. aeruginosa*, a *phoU* mutant displayed increased levels of intracellular guanosine tetraphosphate (ppGpp) and polyphosphate (polyP), impacted antibiotic susceptibility, and decreased growth rate of planktonic bacteria; however, there was no effect on biofilm formation (de Almeida et al., 2015). Two *phoU* homologs from *M. tuberculosis*, *phoY1* and *phoY2*, showed distinct functions in different strains. In the *M. tuberculosis* H37Rv strain, the *phoY2* mutant, but not the *phoY1* mutant, increased the susceptibility to rifampicin and pyrazinamide and decreased persister formation, while in the *M. tuberculosis* Erdman strain, *phoY1* and *phoY2* double mutants, but not the single mutants (deletion of *phoY1* or *phoY2*), increased the susceptibility to rifampicin and decreased persister formation (Shi and Zhang, 2010; Namugenyi et al., 2017). Thus, the biological functions of PhoU homologs show species-specific characteristics, which require individual investigations.

Our previous studies found that in *S. epidermidis*, the biological functions and regulation of PhoU homologs are different from those of some other bacterial species in specific ways (Wang et al., 2017). In *S. epidermidis*, the genome contains two PhoU homologs: *phoU1*, in the same operon as that *pst* and it has high homology with the *phoU* of *E. coli*, and *phoU2*, which is located in the *pit* operon (Wang et al., 2017). PhoU2, but not PhoU1, is an important regulator of biofilm formation and of tolerance to multiple stresses (Wang et al., 2017). The deletion of *phoU2* resulted in growth retardation, decreased persister formation, and biofilm reduction in *S. epidermidis*, while *phoU1* deletion had no effect on the bacterial phenotypes tested (Wang et al., 2017). PhoU2 deletion alters cellular metabolic processes such as inorganic phosphate metabolism, galactose metabolism, the pentose phosphate pathway, and the tricarboxylic acid cycle (Wang et al., 2017). In the genus *Staphylococcus*, both *S. aureus* and *S. epidermidis* are important pathogens, but their pathogenic mechanisms differ. The main pathogenic mechanisms of *S. aureus* are secretions of a variety of toxins to destroy host cells, invasion and survival in cells, and biofilm formation (Fraunholz and Sinha, 2012; Otto, 2014; Moormeier and Bayles, 2017). By comparison, the pathogenesis of *S. epidermidis* is mainly due to the formation of biofilms on materials used for medical interventions that resist clearance by the immune system and antibiotics. Persisters are generated during *S. aureus* or *S. epidermidis* infection (Grassi et al., 2017). A genome analysis of *S. aureus* USA500 2395 allowed for the identification of two *phoU* homologs, *phoU1* and *phoU2*, located in the *pst* operon and *pit* operon, respectively. Overton et al. (2011) analyzed the transcriptomes and the proteomics of the *S. aureus* ATCC 8325 strain and suggested that *phoU1* is involved in persister formation in the presence of the cationic bacitracin (ranalexin) (Overton et al., 2011). The *pit* operon of the *S. aureus* HG003 strain contains the *pitA* and the *phoU2* (*pitR*) genes. A single-point mutation in *pitA* (downstream of *phoU2*) resulted in high tolerance to daptomycin, and *phoU2* (*pitR*) was required for the expression of this phenotype (Mechler et al., 2016). We therefore speculated that the regulatory functions of the two *phoU* homologs (*phoU1* and *phoU2*) of *S. aureus* may differ from those in *S. epidermidis*. In *S. aureus*, the regulation of persister formation and virulence, and their interconnections, may be associated with the *phoU* homologs and requires further investigation.

In the present study, we generated *phoU* single mutants of *S. aureus* strain USA500 2395, named Δ*phoU1* and Δ*phoU2*. The effects of these deletions on bacterial growth, persister formation, and metabolism were investigated. Comparisons of the transcriptome profiles of Δ*phoU1* vs. the parent strain and of Δ*phoU2* vs. the parent strain allowed for differentially expressed genes (DEGs) that were involved in phosphate metabolism, carbon and pyruvate metabolism, and virulence gene expression to be identified. We analyzed intracellular inorganic phosphate (Pi), polyP, glucose, pyruvate, ATP, bacterial survival in cells, and hemolysis, respectively. The deletion of *phoU1* or *phoU2* of *S. aureus* increased carbon metabolism and intracellular ATP levels, which may be associated with the decreased antibiotic tolerance of bacteria and the reduced intracellular survival of bacteria in human lung epithelial A549 cells. The results suggest that both PhoU1 and PhoU2 of *S. aureus* are involved in the regulation of persister generation and virulence.

## Materials and Methods

### Bacterial Strains, Plasmids, Growth Conditions, and Antibiotics

The bacterial strains and plasmids used for cloning are listed in Supplementary Table S1. The *S. aureus* strain USA500 2395 was used for the construction of gene knockout and complementation. *S. aureus* strain USA300 FPR3757 and SA113 were used for gene silencing. *E. coli* DC10B was used for staphylococcal cloning host. *S. aureus* strains were grown at 37°C in tryptic soya broth (TSB) (OXOID, Basingstoke, United Kingdom). *E. coli* was grown at 37°C in Luria broth (1% tryptone, 0.5% NaCl, and 0.5% yeast extract). B2 media (2.5% yeast extract, 1% tryptone, 0.5% glucose, 2.5% NaCl, and 0.1% K_2_HPO_4_) was used for preparing and recovering the electrocompetent cells of *S. aureus* after electroporation. The antibiotics were used at the following concentrations: ampicillin at 100 μg/ml, chloramphenicol at 10 μg/ml, anhydrotetracycline at 50 ng/ml, and erythromycin at 10 μg/ml (Sigma, United States).

### Construction of Gene Knockout, Complementation, and Silencing Strains

The *phoU1* and *phoU2* deletion mutants of *S. aureus* USA500 2395 were constructed using the temperature-sensitive plasmid pKOR1 (Bae and Schneewind, 2006). The upstream and downstream fragments of *phoU1* or *phoU2* were amplified by PCR, ligated by T4 DNA ligase, and cloned into vector pKOR1, resulting in recombinant pKOR1-Δ*phoU1* or pKOR1-Δ*phoU2*. The plasmids pKOR1-Δ*phoU1*andpKOR1-Δ*phoU2* were transferred into *E. coli* DC10Band then into USA500 2395.Δ*phoU1*andΔ*phoU2*were generated by the homologous recombination method of allelic exchange, as described (Bae and Schneewind, 2006). By transferring pKOR1-Δ*phoU2* vector into Δ*phoU1*, the double-deletion mutant of *phoU1* and *phoU2* (Δ*phoU1*Δ*phoU2)* was constructed. The gene deletion mutants were verified by PCR, quantitative reverse transcription-PCR (qRT-PCR), and sequencing.

Complementation of the Δ*phoU1* and the Δ*phoU2*was achieved by the *E. coli*–*Staphylococcus* shuttle vector pCN51 and pRB473. The *phoU1* or the *phoU2*, with their promoter regions, was amplified by PCR and inserted into pCN51. The pCN51-*phoU1* or pCN51-*phoU2* plasmid was transferred into the corresponding deletion mutants by electroporation. The pRB473-*phoU2* plasmid was constructed according to the abovementioned method. The complemented Δ*phoU1*Δ*phoU2* was constructed by transferring the pCN51-*phoU1* and pRB473-*phoU2* by electroporation.

The silencing strains of *phoU1* or *phoU2* were constructed by the shuttle plasmid pMX6 (Helle et al., 2011; Xu et al., 2017). The hairpin structure formed by the plasmid pMX6 was used for constructing antisense RNA (asRNA) expression. The plasmid of asRNA *phoU1* or asRNA *phoU2* was constructed by firstly amplifying a sequence of about 200 nt containing the start codon of the corresponding gene. Then, the sequence was inserted in the reverse direction downstream of the anhydrotetracycline-inducible promoter in pMX6. The plasmid of asRNA *phoU1* or asRNA *phoU2* was transferred into USA500 2395, USA300 FPR3757, and SA113 by electroporation, resulting in the silencing strains. Primers are listed in the Supplementary Tables S3, S4.

### Bacterial Growth Curve and Viable Bacteria Count

*S. aureus* strains were grown to stationary phase (12 h) and then diluted (1:200) in TSB medium. For bacterial growth curves, bacteria were grown at 37°C with shaking at 220 rpm and monitored by measuring the OD_600_ at 1 h intervals for 24 h by Bioscreen C (Turku, Finland). For viable bacterial count, bacteria were grown at 37°C with shaking, and the cells were plated in serial dilutions on TSB agar at 4 and 12 h, then the colony-forming units (CFU) were counted.

### MIC and MBC Determination

According to the CLSI National Committee for Clinical Laboratory, the MICs were determined by using serial twofold dilutions of the antibiotics (vancomycin, levofloxacin, gentamicin, and daptomycin) in Mueller–Hinton broth (MH, OXOID). The initial cell density was 10^5^ CFU/ml. Then, the initial bacteria were inoculated into MH broth for 16–20 h. The MIC is defined as the lowest concentration of antibiotics that inhibited the visible growth of bacteria. MH broth without antibiotic served as the control. The minimal bactericidal concentration (MBC) values were identified by plating 100-μl samples, from tubes with no visible bacterial growth in MIC tests, onto MHB agar plates. The concentration that reduced the viability of the initial bacterial inoculum by =99.9% was the MBC.

### Persister Assay

Persisters were determined as described (Li and Zhang, 2007). *S. aureus* strains were grown in TSB for 12 h to reach the stationary phase. Different 25 × MIC antibiotics were added to the cultures (final concentrations: levofloxacin at 12.5 mg/L and vancomycin at 25 mg/L). At each time point (0, 4, 8, 12, 24, 48, and 72 h), 1 ml of bacteria was collected by centrifugation (6,000 rpm), washed twice with cold saline, serially diluted 10-fold, and plated on TSB agar. Then, the CFU were counted.

### Sensitivity to H_2_O_2_ and SDS

Overnight cultures (12 h) of *S. aureus* strains were diluted 1:200 into 7 mM H_2_O_2_ or 0.005% sodium dodecyl sulfate (SDS) TSB and incubated at 37°C, and the OD_600_ was measured. Overnight cultures of *S. aureus* strains were serially diluted 10-fold. Five microliters of the diluted samples was spotted onto TSA plates containing 7 mM H_2_O_2_ or 0.005% SDS and incubated at 37°C overnight. The plates with bacterial colonies were photographed.

### RNA Extracting and Sequencing

Total RNA for RNA-Seq and qRT-PCR was extracted by RNeasy Mini kit (QIAGEN, Hilden, Germany) following the manufacturer’s instructions. In brief, *S. aureus* and the derivative strains were diluted 1:200 into 20 mM TSB and incubated at 37°C. At 12 h, 8 ml of bacteria was collected at 6,000 rpm and washed twice times with cold saline. With 0.5 ml of 0.1 mm zirconia-silica beads, the cells were homogenized for five rounds using a Mini-Bead beater (Biospec, Bartlesville, OK, United States) at 4,800 rpm for 1 min and were cooled on ice for 1 min. Then, the samples were centrifuged at 12,000 rpm, and RNA in the supernatant was extracted using the silica-based filter of Neasy Mini kit.

The samples were prepared according to the Illumina RNA Sequencing Sample Preparation Guide. In brief, three biological replicates for each of the *S. aureus* were treated with RNase-free DNase I (Takara) to remove the genomic DNA. The BioAnalyzer 2100 system was used to evaluate RNA quality. The samples were treated with the RiboZero rRNA removal kit (gram-positive organisms) to remove ribosomal RNA. Fragmented RNA was reverse-transcribed using random primers. The cDNA library included fragment sizes of 200–300 bp, which were prepared by the mRNA-Seq Sample Prep kit and verified on the BioAnalyzer 2100 system. Then, the fragment size is amplified by Illumina cBot and sequenced by Illumina HiSeq 2500.

### RNA-Seq Data Analysis and qRT-PCR Validation

Quality control involves discarding of rRNA reads, sequencing adapters, short fragments, and other low-quality reads. The remaining reads were multi-mapped to the genome of *S. aureus* USA500 2395 at the NCBI website with the Bowtie2 software. BED Tools software was used to count the transcript expression levels. Per kilobase of gene per million mapped reads (RPKM) reported the RNA-seq gene expression values. Integrated Genomics Viewer was used to visualize the date. DEGseq software was used to quantify the differential expression of different transcripts. Significant differences in expression ratios were defined as at least 2.0- or 0.5-fold change in transcript level. The *P*-values cutoff was calculated (0.05). IPA Software was used to analyze differentially expressed genes in the canonical pathway. The number of genes mapped to the pathway vs. the total genes present in the canonical pathway determined the significance of the pathway.

A pool of 5 μg of total RNA for *S. aureus* strains was DNA-digested and reverse-transcribed into cDNA using PrimeScript TM RT reagent kit. Then, 100 ng/μl cDNA was used for qRT-PCR with TB green PCR reagents. All reagents were from Takara Biotechnology. The reactions were performed in a Mastercycler realplex system (Eppendorf AG, Hamburg, Germany) and normalized using *gyrB* (DNA gyrase subunit B) as the housekeeping gene. Each gene qRT-PCR was performed in triplicate.

### Protein–Protein Interaction Network

Cytoscape software was used to construct a protein–protein interaction network (PPI) according to the Kyoto Encyclopedia of Genes and Genomes (KEGG) database^1^.

### Inorganic Phosphate Determination

Intracellular Pi was quantified with a commercially available kit (no. ab65622; Abcam), which was modified for *S. aureus* (Mechler et al., 2015). In brief, overnight cultures of *S. aureus* strains were diluted 1:200 into TSB and incubated at 37°C. After 12 h, the cells were harvested by centrifugation at 4,000 rpm for 10 min at 4°C. The cells were washed twice with ice-cold, double-distilled water and adjusted to OD600 ≈1; then, they were lysed with 0.1 mm glass-silica beads in a BeadBeater apparatus (BioSpec), followed by centrifugation to get the supernatant. The supernatant was used to measure OD_650_ (Thermo VARIOSKAN LUX). The Pi levels were determined according to the manufacturer’s instructions.

### Polyphosphate Determination

Intracellular polyP levels were determined using 4’-6-diamidino-2-phenylindole (DAPI; Sigma) as described previously (Aschar-Sobbi et al., 2008). Overnight cultures of *S. aureus* strains were diluted 1:200 into TSB and incubated at 37°C. After 12 h, the cells were washed twice with Tris-HCl buffer (100 mM Tris, pH 7.4) and adjusted to OD_600_ ≈ 1. DAPI was added to a final concentration of 20 μM. After 15 min of agitation at room temperature in the dark, the fluorescence signal was determined using a microplate reader with excitation at 415 nm and emission at 550 nm (Thermo VARIOSKAN LUX).

### Glucose and Pyruvate Detection

Glucose and pyruvate were measured using the Glucose Assay Kit (ab65333; Abcam) and Pyruvate Assay Kit (ab65342; Abcam) according to the manufacturer’s instructions (Liang et al., 2018; Schnack et al., 2019). *S. aureus* strains were diluted 1:200 into TSB and incubated at 37°C. After 12 h, culture supernatant and cells were harvested. The cells were washed twice with phosphate-buffered saline (PBS) and adjusted to OD_600_ ≈ 1 with assay buffer and then were lysed with 0.1-mm glass-silica beads in a BeadBeater apparatus (BioSpec), followed by centrifugation to obtain the supernatant. The fluorescence signal of the supernatant was determined using a microplate reader with excitation at 535 nm and emission at 587 nm (Thermo VARIOSKAN LUX).

### Intracellular ATP Detection

The ATP levels of *S. aureus* strains were measured using a Promega BacTiter Glo kit according to the manufacturer’s instructions (Bonora et al., 2015). *S. aureus* strains were diluted 1:200 into TSB and incubated at 37°C. After 12 h, the cells were washed twice with PBS and adjusted to OD_600_ ≈1. A volume of BacTiter-Glo^TM^ Reagent was added equal to the volume of the cell culture medium present in each well. The complexes were mixed briefly and incubated for 5 min. Luminescence was determined using a microplate reader (Thermo VARIOSKAN LUX).

### Invasion and Intracellular Survival Assays

An assay of bacterial invasion was performed as previously described (Liang et al., 2006; Sharma et al., 2013; Mechler et al., 2015). A549 human lung epithelial cells were cultured in Dulbecco’s modified Eagle’s medium/F-12 medium supplemented with 10% fetal calf serum, streptomycin (100 μg/ml), and penicillin (100 μg/ml) in a 5% CO_2_ incubator at 37°C. The cells were passaged and expanded every 2 days. At 1 day prior to infection, 2 × 10^5^ cells were seeded in 24-well plates with an antibiotic-free cell culture medium and incubated at 37°C in a 5% CO_2_ incubator. *S. aureus* strains were grown for 4 and 12 h in TSB. One milliliter of the bacterial culture was washed with ice-cold saline and resuspended in 1 ml of cell culture medium. Approximately 2 × 10^6^ CFU/ml of *S. aureus* strains were seeded in a 24-well plate. The plates were centrifuged at 1,000 rpm for 5 min to synchronize infection and then incubated for 1 h at 37°C in a 5% CO_2_ incubator. The culture medium was removed. Extracellular bacteria were treated with 100 μg/ml gentamicin and 20 μg/ml lysostaphin (Sangon Biotech) for 30 min. The monolayer cells were washed three times with PBS (pH 7.4) and incubated for an additional 1 h (invasion capacity) or 24 h (intracellular survival). Intracellular bacteria were counted for the CFU by lysis of the host cells with 0.01% Triton X-100.

### Rabbit Erythrocyte Lysis Assay

As rabbit erythrocytes were exquisitely sensitive to alpha-toxin (Bernheimer et al., 1968), assessment of the alpha-hemolysin activity was done by the analysis of rabbit erythrocytes lysis as Kernodle DS et al. had previously described (Sharma-Kuinkel et al., 2012). Briefly, the *S. aureus* strains were diluted 1:200 into TSB and incubated at 37°C. After 12 h, the culture supernatant was harvested. The supernatant was removed with a 0.22 μm filter (Millipore). Commercial 4% rabbit erythrocytes (SBJ-RBC-RAB003, Sbjbio, China) stored in Alsevers solution were four times diluted with PBS. The supernatant was added, equal to the volume of 1% of rabbit erythrocytes, and then incubated at 37°C for 30 min. The OD_550_ of each well was measured by a spectrophotometer. Then, 0.1% Triton X-100 served as the 100% hemolysis control (positive control), and PBS was the negative control. All experiments were performed in triplicate.

### Extracellular Alpha-Hemolysin Western Blot Assay

The collected supernatant was added 5 × loading buffer and the mixture was heated at 100°C for 5 min. Equal volumes of the mixture were separated using SDS-PAGE (10%) and transferred to polyvinylidene fluoride membrane (pore size, 0.45 μm; Millipore) by electrotransfer. The membranes were blocked with 5% skim milk for 2 h at room temperature and then incubated with alpha-hemolysin polyclonal antibody (Sigma S7531) overnight at 4°C. After washing three times with PBST, the membranes were incubated with HRP-conjugated goat anti-rabbit IgG (Santa Cruz, Santa Cruz, CA, United States). The immunoreactive bands were detected by visualization using an enhanced chemiluminescence Western blotting system (Thermo Fisher Scientific, Waltham, MA, United States).

### Statistical Analysis

All of the data were analyzed with SPSS (version 16.0) and compared using the independent-samples *t*-test. Differences with *P*-value < 0.05 were considered as statistically significant.

### RNA-Seq Data Accession Number

The RNA-Seq data were submitted to the Gene Expression Omnibus database. The accession number was GSE139071.

## Results

### Construction of *phoU1* and *phoU2* Deletion Mutant Strains

In the genome of *S. aureus*, strain USA500 2395 (GenBank accession number CP007499), two PhoU homologs are present: *phoU1* is located in the *pst* operon and *phoU2* is located in the *pit* operon. An alignment analysis of the PhoU homologs showed a high identity at the nucleotide level (>99%) and at the amino acid level (100%) in *S. aureus* strains (Supplementary Table S1). The identity of *S. aureus* PhoU1 or PhoU2, when compared with *S. epidermidis*, was 70 and 87%, respectively, at the nucleotide level and 95% for both proteins at the amino acid level (Supplementary Table S1).

Single or double mutants of *phoU1* and *phoU2* were constructed in the USA500 2395 strain using the temperature-sensitive plasmid pKOR1 and were named Δ*phoU1*, Δ*phoU2*, and Δ*phoU1*Δ*phoU2*. The mutants were verified by PCR, qRT-PCR, and sequencing (the data not shown). Complementation of the Δ*phoU1* and the Δ*phoU2* mutants was achieved using the vectors pCN51 and pRB473, which were named C-Δ*phoU1* and C-Δ*phoU2*, respectively. We then determined the growth curve and the viable bacterial counts of the 3 *phoU* mutants and the wild-type strain USA500 2395. Both the growth curve and the viable bacterial count of Δ*phoU1* or Δ*phoU2* were similar to USA500 2395 (Supplementary Figures S1A,B).

### Antibiotic Tolerance of the *phoU1* and *phoU2* Deletion Mutants

Previous reports indicated that PhoU2, but not PhoU1, impacted antibiotic tolerance in *S. epidermidis* (Wang et al., 2017); therefore, we investigated the effects of deleting the *phoU* homolog on *S. aureus* antibiotic tolerance. The antibiotic tolerance of Δ*phoU1*, Δ*pho*U2, and USA500 2395 was determined using a modified procedure described by Li and Zhang. The Δ*phoU1*, Δ*pho*U2, and USA500 2395 strains were incubated in TSB for 12 h (to reach stationary growth phase), followed by 5 days of incubation with 25 × MIC vancomycin (25 μg/ml) or levofloxacin (12.5 μg/ml). The surviving CFU were counted at different time points. The antibiotic tolerance of both Δ*phoU1* and Δ*phoU2* showed 4-log reductions when compared with USA500 2395, and no viable bacteria were detected in either Δ*phoU1* or Δ*phoU2* after vancomycin exposure with 25 × MIC for 48 h, whereas in USA500 2395, 3.8 × 10^4^ CFU were detected (Table 1). Moreover, no viable bacteria were detected in either Δ*phoU1* or Δ*phoU2* after exposure to 25 × MIC levofloxacin for 24 h, compared to USA500 2395 where 5 × 10^4^ CFU were detected (Table 1). The complemented strains C-*phoU1* and C-*phoU2* restored the antibiotic tolerance, while the P-*phoU1* and P-*phoU2* strains with empty plasmids performed the same as the *phoU* mutants. The MIC and MBC of Δ*phoU1* and Δ*phoU2* were similar to that of USA500 2395 (Supplementary Table S2).

**TABLE 1:** Deletion of *phoU1* or *phoU2* decreased the persister formation in *Staphylococcus aureus* under the pressure of antibiotics.

Antibiotic/bacterial	Time point	CFU/ml						
		USA500 2395	Δ *phoU1*	P-Δ *phoU1*	C-Δ *phoU1*	Δ *phoU2*	P-Δ *phoU2*	C-Δ *phoU2*
Van	Start	2.0 × 10^10^	1.8 × 10^10^	1.6 × 10^10^	1.8 × 10^10^	2.0 × 10^10^	1.6 × 10^10^	1.8 × 10^10^
	4 h	8.0 × 10^8^	6.3 × 10^8^	7.1 × 10^8^	6.8 × 10^8^	8.0 × 10^8^	8.0 × 10^8^	6.0 × 10^8^
	8 h	1.4 × 10^8^	2.0 × 10^7^	4.6 × 10^7^	5.8 × 10^7^	6.0 × 10^7^	8.0 × 10^7^	8.0 × 10^7^
	12 h	1.0 × 10^8^	2.5 × 10^7^	4.3 × 10^7^	9.2 × 10^7^	2.0 × 10^7^	4.0 × 10^7^	1.4 × 10^8^
	24 h	1.2 × 10^6^	3.0 × 10^5^	4.0 × 10^5^	6.2 × 10^6^	8.0 × 10^5^	1.2 × 10^6^	4.0 × 10^7^
	48 h	3.8 × 10^4^	0	0	2.3 × 10^5^	0	0	8.0 × 10^5^
	72 h	4.0 × 10^3^	0	0	4.0 × 10^3^	0	0	4.0 × 10^3^

Lev	Start	2.0 × 10^10^	1.8 × 10^10^	1.6 × 10^10^	1.8 × 10^10^	2.0 × 10^10^	1.6 × 10^10^	1.8 × 10^10^
	4 h	1.0 × 10^7^	2.0 × 10^7^	1.5 × 10^7^	2.3 × 10^7^	1.4 × 10^7^	2 × 10^6^	2.2 × 10^7^
	8 h	1.6 × 10^5^	6.9 × 10^4^	9.7 × 10^4^	2.7 × 10^5^	8 × 10^4^	1.2 × 10^5^	1.6 × 10^5^
	12 h	1.2 × 10^5^	8 × 10^4^	1.4 × 10^5^	1.4 × 10^5^	1.0 × 10^4^	1.4 × 10^4^	1.4 × 10^5^
	24 h	5 × 10^4^	0	0	2 × 10^4^	0	0	0
	48 h	0	0	0	0	0	0	0
	72 h	0	0	0	0	0	0	0

*Overnight cultures (12 h) of ΔphoU1, ΔphoU2, and USA500 2395 were added into tryptic soya broth medium containing 25 × MIC of vancomycin (Van, 25 μg/ml) or levofloxacin (Lev, 12.5 μg/ml) and incubated at 37°C until 72 h. The colony-forming units of the above strains were performed at the indicated time points. The complementation of ΔphoU1 or ΔphoU2 mutant was abbreviated as C-phoU1 or C-phoU2, which were transferred as pCN51-phoU1 or pCN51-phoU2 into the corresponding deletion mutants. The empty vectors of pCN51 transferred into the ΔphoU1 or ΔphoU2 mutants were named P-phoU1 or P-phoU2.*

### Tolerance to H_2_O_2_ and SDS o*f phoU1* and *phoU2* Deletion Mutants

**FIGURE 1:**
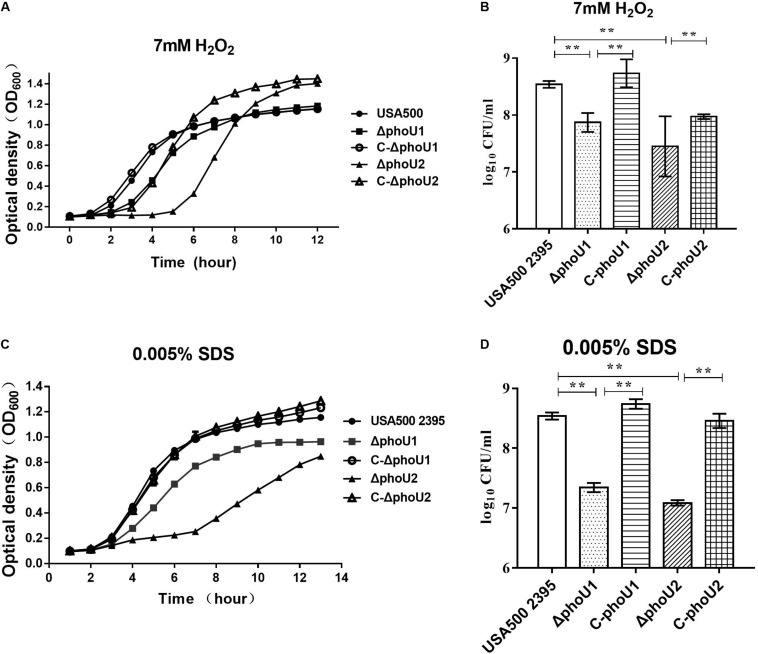
Deletion of *phoU1* or *phoU2* increased the sensitivity to H_2_O_2_ and sodium dodecyl sulfate (SDS) of *Staphylococcus aureus*. Overnight cultures (12 h) of Δ*phoU1*, Δ*phoU2*, C- *phoU1*, C- *phoU2*, and USA500 2395 strains were diluted at 1:200 into tryptic soya broth (TSB) containing either **(A)** hydrogen peroxide (H_2_O_2_; 7 mM) or **(C)** SDS (0.005%), then grown at 37°C with shaking at 220 rpm, and monitored by measuring the OD_600_ at the indicated time points until 12 h. **(B)** H_2_O_2_ (7 mM) or **(D)** SDS (0.005%), collecting **(A,C)** 6 h culture; bacteria in serial dilutions were plated on TSB agar plates and colony-forming units were counted. The experiments were repeated three times, and error bars indicate the standard deviation. Mutant strains exhibited significant differences (**P < 0.01) when compared with the wild-type and the complemented strains C-*phoU1* or C-*phoU2.*

We evaluated the tolerance of Δ*phoU1* and Δ*phoU2* to stresses (SDS and H_2_O_2_) and found that, when exposed in TSB containing 7 mmol H_2_O_2_, the lag phase of Δ*phoU1* and Δ*phoU2* was extended by 2 and 4 h, respectively, in comparison to USA500 2395 (Figure 1A). When cultured in 0.005% SDS TSB for 12 h, the OD_600_ values of Δ*phoU1* and Δ*phoU2* were 0.96 ± 0.013 and 0.84 ± 0.007, respectively, which were all lower than that of USA500 2395 (1.15 ± 0.023) (Figure 1C). When cultured in 7 mmol H_2_O_2_ (or 0.005% SDS) TSB for 6 h, bacteria were collected and plated on TSB agar plates in serial dilutions, and CFU were counted. The CFU of Δ*phoU1* and Δ*phoU2* were log_10_ 7.92 ± 1.74 (or log_10_ 7.6 ± 0.82) and log_10_ 7.48 ± 0.53 (or log_10_ 7. 20 ± 0.43), respectively, which were all lower than that of USA500 2395 log_10_ 8.6 ± 0.72 (or log_10_ 8.5 ± 0.53) (Figures 1B,D). The complemented strains C-*phoU1* and C-*phoU2* restored the tolerance to H_2_O_2_ and SDS.

The Δ*phoU1*, Δ*pho*U2, and USA500 2395 strains were exposed onto TSB agar containing either H_2_O_2_ (7 mmol) or SDS (0.005%) and then incubated for 24 h at 37°C. The number of colonies on the plates displayed the final impact on the growth under the pressure of H_2_O_2_ and SDS. The Δ*phoU1* and Δ*phoU2* mutants displayed higher sensitivity to SDS than that of the parent strain, when 10^3^ CFU was spotted onto TSB agar containing SDS (0.005%) (Supplementary Figure S4). However, the sensitivity of the Δ*phoU1* and Δ*phoU2* mutants to H_2_O_2_ was similar to that of the parent strain (Supplementary Figure S4).

### Comparison of the Transcriptomes of Δ*phoU1*, ΔphoU2, and USA500 2395

**FIGURE 2:**
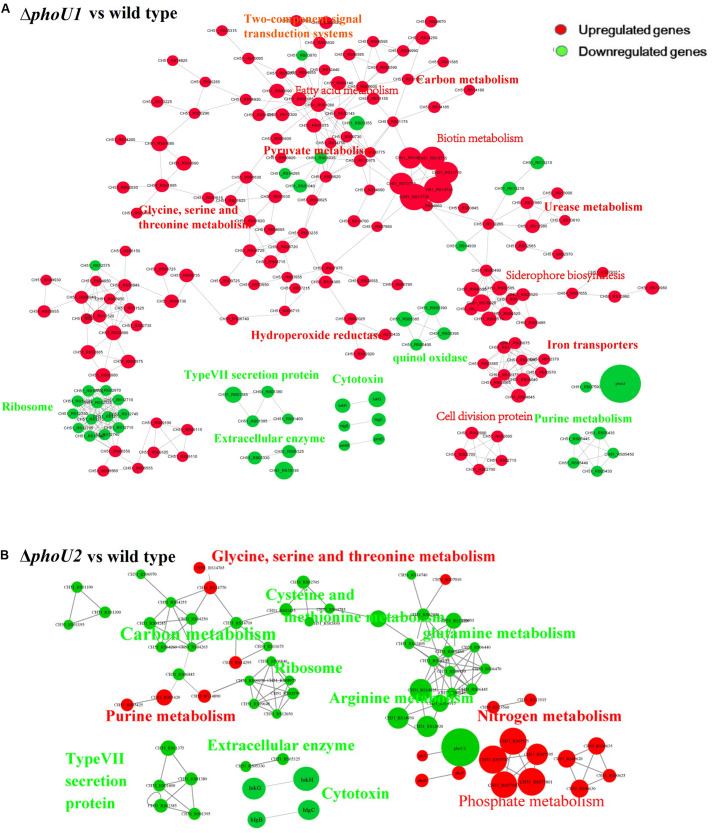
Protein–protein interaction network. Up-regulated or down-regulated differentially expressed genes are indicated in red or green, respectively. The size of the node indicates multiple gene expression. The lines represent protein–protein interactions, including binding/association, phosphorylation, activation, and inhibition. Interaction networks of proteins encoded by between DEGs identified by transcriptome comparison between Δ*phoU1* and wild type **(A)** and Δ*phoU2* and wild type **(B)**.

For the analysis of Δ*phoU1*, Δ*pho*U2, and USA500 2395 transcriptomes, bacterial RNA was extracted at the stationary phase (after 12 h of growth) and analyzed by RNA-Seq. Transcriptome analysis indicated 573 differentially expressed genes between Δ*pho*U1 and USA500 2395, including 456 up-regulated and 117 down-regulated genes. Between Δ*pho*U2 and USA500 2395, 285 differentially expressed genes were identified, including 53 up-regulated and 232 down-regulated genes. We selected 65 DEGs for validation by RT-qPCR and established that 61 DEGs were consistent, following RNA-Seq. A PPI was constructed based on the KEGG database^1^. This suggested that the DEGs between Δ*pho*U1 and USA500 2395 were widely involved in various metabolic pathways (including iron transport and the metabolism of carbon, pyruvate, urease, and fatty acids) and that a majority of these genes were up-regulated (Table 2 and Figure 2). Furthermore, the transcription levels of the DEGs between Δ*pho*U2 and USA500 2395 that were linked to phosphate metabolism were up-regulated, whereas the majority of those linked to carbon and pyruvate metabolism were down-regulated (Table 3 and Figure 2). In both Δ*phoU1* and Δ*phoU2* mutants, the transcription levels of virulence-related factors (including the type VII secretion system operon *esxA*, *esaA*, *essA*, *essB*, and *essC*), serine-like proteinases (*sspA*, *splF*, *splE*, *splD*, *splC*, *splB*, and *splA*), leukocidin (*lukG*, *lukH*, and *lukD*), and hemolysin (*hlgA*, *hlgC*, *hlgB*, and *hl*α) were down-regulated compared with those of USA500 2395 (Supplementary Table S5). The transcription levels of fibrinogen-binding genes and *sarA* (a regulator of fibronectin-binding proteins, hemolysins, and serine proteases) between Δ*pho*U1 and USA500 2395 were down-regulated, whereas those of *codY* (a repressor of the Agr system) and *graS*/*graR* were up-regulated. The transcription levels of *phoP*/*phoR* between Δ*pho*U2 and USA500 2395 were up-regulated, while those of *sarA*, *sarR*, *sarZ*, *codY*, and *rot* were down-regulated.

**TABLE 2:** Pathway analysis of differentially expressed genes of Δ*phoU1* and USA500 2395.

Gene	Description	Fold change	
		RNA-seq	qRT-PCR
**Carbon metabolism**			
*CH51_RS01175*	Sorbitol dehydrogenase	2.04	ND
*CH51_RS14180*	Gluconate permease	2.17	ND
*CH51_RS14185*	Gluconokinase	2.52	ND
*CH51_RS14680*	Acyl esterase	2.26	ND
*CH51_RS14690*	Pantoate-beta-alanine ligase	2.14	ND
*CH51_RS14700*	2-Dehydropantoate 2-reductase	2.63	ND
*CH51_RS11580*	Carbohydrate kinase	2.33	ND
*CH51_RS11585*	Sucrose-6-phosphate hydrolase	2.17	ND
*CH51_RS06555*	Ribulose-phosphate 3-epimerase	2.16	ND
*CH51_RS08720*	Glycine dehydrogenase	2.94	ND
*CH51_RS02915*	Serine acetyltransferase	2.52	ND
*CH51_RS00820*	Formate dehydrogenase	2.06	ND
*CH51_RS09725*	Formate-tetrahydrofolate ligase	2.07	ND
*CH51_RS05415*	Bifunctional methylenetetrahydrofolate dehydrogenase/methenyltetrahydrofolate cyclohydrolase	2.04	ND
*CH51_RS08715*	Glycine dehydrogenase	3.39	4.21 ± 0.83
**Pyruvate metabolism**			
*CH51_RS03145*	Acetyl-CoA acetyltransferase	2.58	ND
*CH51_RS00775*	Aldehyde dehydrogenase	2.02	ND
*CH51_RS01035*	Formate acetyltransferase	0.37	0.52 ± 0.12
*CH51_RS01095*	Acyl CoA:acetate/3-ketoacid CoA transferase	2.74	ND
*CH51_RS10975*	Aldehyde dehydrogenase	2.84	ND
*CH51_RS14295*	Lactate dehydrogenase	0.43	ND
*CH51_RS04930*	CoA-disulfide reductase	2.32	ND
*CH51_RS09730*	Acetate-CoA ligase	2.31	ND
*CH51_RS14750*	Acetyl-CoA synthetase	2.17	1.91 ± 0.61
**Glycolysis/gluconeogenesis**			
*CH51_RS03355*	Zinc-dependent alcohol dehydrogenase	0.24	ND
*CH51_RS10975*	Aldehyde dehydrogenase	2.84	ND
*CH51_RS09730*	Acetate-CoA ligase	2.31	ND
*CH51_RS14750*	Acetyl-CoA synthetase	2.17	ND
*CH51_RS00775*	Aldehyde dehydrogenase	2.02	ND
**Propanoate metabolism**			
*CH51_RS08625*	Alpha-ketoacid dehydrogenase subunit beta	2.2	ND
*CH51_RS08620*	2-Oxo acid dehydrogenase subunit E2	2.44	ND
**Glyoxylate and dicarboxylate metabolism**			
*CH51_RS08725*	Aminomethyltransferase	2.97	ND
*CH51_RS03045*	HAD family hydrolase	2.01	ND
**Purine metabolism**			
*CH51_RS06550*	GTPase	2.52	ND
*CH51_RS05100*	GTP pyrophosphokinase	2.13	ND
*CH51_RS05450*	Amidophosphoribosyl transferase	0.46	ND
*CH51_RS06795*	DNA polymerase III subunit alpha	2.34	ND
*purE*	Phosphoribosylaminoimidazole synthase	0.45	ND
*purS*	Phosphoribosylformylglycinamidine synthase subunit	0.33	ND
*purQ*	Phosphoribosylformyl glycinamidine synthase subunit	0.43	ND
*purL*	phosphoribosylformylglycinamidine synthase subunit	0.41	ND
*CH51_RS09215*	Bifunctional (p)ppGpp synthetase/guanosine-3\’,5\’-bis(diphosphate) 3\’-pyrophosphohydrolase	2.16	ND
*CH51_RS09555*	DNA polymerase III subunit alpha	2.42	ND
*CH51_RS02730*	Hypoxanthine-guanine phosphoribosyltransferase	2.35	ND
**Urease metabolism**			
*CH51_RS12985*	Urease subunit beta	3.58	2.82 ± 0.62
*CH51_RS12980*	Urease subunit gamma	2.86	1.61 ± 0.37
*CH51_RS12975*	Urea transporter	2.06	ND
*CH51_RS13000*	Urease accessory protein UreF	2.45	ND
*CH51_RS13010*	Urease accessory protein	2.1	ND
**Iron metabolism**			
*fhuD*	Iron-dicitrate ABC transporter permease	2.25	ND
*fhuB*	Iron ABC transporter permease	2.47	ND
*fhuC*	ABC transporter substrate-binding protein	2.91	ND
*CH51_RS03385*	Iron ABC transporter permease	2.17	ND
*CH51_RS03560*	Iron ABC transporter permease	2.13	ND
*CH51_RS03565*	Iron ABC transporter permease	2.28	ND
*CH51_RS03570*	Iron ABC transporter permease	2.32	ND
*CH51_RS04045*	Iron-dicitrate ABC transporter	2.25	ND
*CH51_RS04040*	Iron ABC transporter ATP-binding protein	2.11	ND
*sbnA*	Siderophore biosynthesis protein	2.73	1.78 ± 0.14
*sbnB*	2,3-Diaminopropionate biosynthesis protein	2.59	ND
*sbnC*	IucA/IucC family siderophore biosynthesis protein	2.98	ND
*CH51_RS00500*	Staphyloferrin B export MFS transporter	4.38	ND
*sbnE*	Staphyloferrin B biosynthesis enzyme	4.2	ND
*sbnF*	Staphyloferrin B biosynthesis protein	4.21	ND
*sbnG*	Siderophore biosynthesis protein	4.1	ND
*sbnH*	Staphyloferrin B biosynthesis decarboxylase	3.09	ND
*sbnI*	Siderophore biosynthesis protein	3.49	1.62 ± 0.51
*CH51_RS00745*	Iron-regulated surface sugar transferase	2.3	ND
*CH51_RS04775*	Iron-sulfur cluster assembly accessory protein	2.03	ND
*CH51_RS09715*	Iron-regulated surface determinant protein H	5.38	ND
*CH51_RS06055*	Iron-regulated surface determinant protein B	3.06	ND
*CH51_RS06075*	Heme uptake system protein IsdE	2.67	ND
**Phosphate metabolism**			
*phoU*	Phosphate transport system regulatory protein	0	ND
*pstC*	Phosphate ABC transporter permease subunit	0.45	1.85 ± 0.16
**Fatty acid metabolism**			
*CH51_RS01075*	Acetyl-CoA C-acyltransferase	3.91	ND
*CH51_RS01080*	3-Hydroxyacyl-CoA dehydrogenase	4.12	ND
*CH51_RS01085*	Glutaryl-CoA dehydrogenase	3.86	ND
*CH51_RS01090*	Long-chain fatty acid-CoA ligase	4.2	ND
*fapR*	Transcription factor FapR	2.51	ND
*CH51_RS06590*	Phosphate acyltransferase	2.73	ND
*CH51_RS06595*	Malonyl CoA-ACP transacylase	2.3	ND
*CH51_RS06600*	3-Oxoacyl	2.4	ND
*CH51_RS04990*	3-Oxoacyl-ACP synthase III	2.44	ND
*CH51_RS03140*	Long-chain-fatty-acid-CoA ligase	3.85	ND
*CH51_RS05715*	Glycerophosphodiester phosphodiesterase	2.04	ND
*CH51_RS07225*	Cardiolipin synthase	2.19	ND
*CH51_RS03620*	NAD(P)-dependent oxidoreductase	2.95	ND
*CH51_RS03940*	LTA synthase family protein	2.01	ND
**Biotin metabolism**			
*CH51_RS13710*	Hypothetical protein	14.6	ND
*CH51_RS13715*	6-Carboxyhexanoate-CoA ligase	19.84	ND
*CH51_RS13720*	Aminotransferase class V-fold PLP-dependent enzyme	13.9	ND
*CH51_RS13725*	Biotin synthase	15.64	6.8 ± 0.81
*CH51_RS13730*	Adenosylmethionine-8-amino-7-oxononanoate aminotransferase BioA	38.04	10.36 ± 1.23
*CH51_RS13735*	Dethiobiotin synthase	35.98	ND
*CH51_RS13760*	Hypothetical protein	16.1	ND
*CH51_RS07980*	Biotin	2.03	ND
*CH51_RS12955*	Biotin transporter BioY	6.42	ND
**Proline metabolism**			
*CH51_RS00845*	Acetylglutamate kinase	2.01	ND
*CH51_RS04860*	Ornithine aminotransferase 2	2.15	ND
*CH51_RS16020*	Hypothetical protein	7.01	ND
*CH51_RS08555*	Pyrroline-5-carboxylate reductase	2.49	ND
**Lysine biosynthesis**			
*CH51_RS07635*	4-Hydroxy-tetrahydrodipicolinate reductase	2.12	ND
*CH51_RS07655*	Diaminopimelate decarboxylase	2.37	ND
*CH51_RS13980*	DUF2338 domain-containing protein	3.26	ND
*CH51_RS13990*	Diaminopimelate epimerase	2.69	ND
*CH51_RS07275*	Homoserine dehydrogenase	2.01	ND
**Glycine, serine, and threonine metabolism**			
*CH51_RS01330*	Glycine-glycine endopeptidase LytM	3.59	ND
*CH51_RS07525*	Glycine glycyltransferase FemB	2.14	ND
*CH51_RS01620*	Glycine cleavage system protein H	2.57	ND
*CH51_RS01625*	Hypothetical protein	2.32	ND
*CH51_RS01630*	Deacetylase SIR2	2.86	ND
*CH51_RS01635*	Lipoate-protein ligase A	3.03	ND
*CH51_RS09640*	GAF domain-containing protein	2.16	ND
**Cysteine, histidine, and methionine metabolism**			
*CH51_RS00060*	Homoserine O-acetyltransferaseisomerase	2.2	ND
*CH51_RS02430*	Cysteine synthase family protein	2.16	ND
*CH51_RS13215*	Urocanate hydratase	0.31	ND
*CH51_RS13210*	Imidazolonepropionase	0.28	ND

*ND, not done.*

**TABLE 3:** Pathway analysis of differentially expressed genes of Δ*phoU2* and USA500 2395.

Gene	Description	Fold change	
		RNA-seq	qRT-PCR
**Carbon metabolism**			
*CH51_RS04260*	2,3-Bisphosphoglycerate-independent phosphoglycerate mutase	0.42	*ND*
*CH51_RS04265*	Enolase	0.49	*ND*
*CH51_RS04255*	Triose-phosphate isomerase	0.42	*ND*
*CH51_RS02745*	Cysteine synthase	0.31	*ND*
*CH51_RS04250*	Phosphoglycerate kinase	0.36	*ND*
*CH51_RS14740*	Malate:quinone oxidoreductase	0.49	*ND*
*CH51_RS04245*	Aldehyde dehydrogenase	0.34	*ND*
**Pyruvate metabolism**			
*CH51_RS14295*	D-Lactate dehydrogenase	3.11	*ND*
*CH51_RS01035*	Pyruvate formate lyase	2.71	*ND*
*CH51_RS14750*	2-Isopropylmalate synthase	0.49	*ND*
**Glycolysis/gluconeogenesis**			
*CH51_RS03355*	Alcohol dehydrogenase	3.35	*ND*
**Pentose and glucuronate interconversions**			
*CH51_RS01190*	2-C-Methyl-D-erythritol 4-phosphate cytidylyltransferase	0.39	*ND*
*CH51_RS01195*	Alcohol dehydrogenase	0.47	*ND*
**Purine metabolism**			
*CH51_RS05420*	5-(carboxyamino)imidazole ribonucleotide mutase	4.99	*ND*
*CH51_RS06845*	Polyribonucleotide nucleotidyltransferase	0.35	*ND*
*CH51_RS05425*	5-(carboxyamino)imidazole ribonucleotide synthase	3.06	*ND*
**Iron metabolism**			
*CH51_RS00480*	Iron-siderophore ABC transporter substrate-binding protein	0.41	*ND*
*CH51_RS06060*	Iron-regulated surface determinant protein A	0.23	*ND*
*CH51_RS06065*	Iron-regulated surface determinant protein C	0.44	*ND*
**Nitrogen metabolism**			
*CH51_RS13560*	Nitrate reductase subunit alpha	3.1	*ND*
**Phosphate metabolism**			
*phoU1*	Phosphate transport system regulatory protein	22.2	35.56 ± 5.65
*pstB*	Phosphate ABC transporter ATP-binding protein	24.55	32.37 ± 4.8
*pstA*	Phosphate ABC transporter, permease protein	26.05	19.74 ± 2.7
*pstC*	Phosphate ABC transporter permease subunit	20.97	46.05 ± 6.73
*pstS*	Phosphate-binding protein	10.23	25.14 ± 6.21
*phnE*	Phosphonate ABC transporter, permease protein	6.19	*ND*
*phnF*	Phosphonate ABC transporter, permease protein	3.47	*ND*
*phnC*	Phosphonates import ATP-binding protein	6.38	*ND*
*phnD*	Phosphate/phosphite/phosphonate ABC transporter substrate-binding protein	5.81	*ND*
*phoA*	Alkaline phosphatase	3.28	1.91 ± 0.15
*phoU2*	DUF47 domain-containing protein	0.04	
*pitA*	Inorganic phosphate transporter	2.52	2.83 ± 0.12
*phoR*	Sensor histidine kinase	3.64	2.48 ± 0.51
*phoP*	DNA-binding response regulator	3.42	*ND*
**Cysteine and methionine metabolism**			
*CH51_RS09640*	GAF domain-containing protein	0.48	*ND*
*CH51_RS10035*	S-Adenosylmethionine synthase	0.39	*ND*
*CH51_RS02435*	Cystathionine gamma-synthase	0.32	*ND*
*CH51_RS02430*	Cysteine synthase family protein	0.5	*ND*
**Alanine and aspartate metabolism**			
*CH51_RS06445*	Aspartate carbamoyltransferase	0.36	*ND*
*CH51_RS06460*	Carbamoyl-phosphate synthase large chain	0.37	*ND*
*CH51_RS06455*	Carbamoyl-phosphate synthase small chain	0.38	*ND*
**Glycine, serine, and threonine metabolism**			
*CH51_RS14765*	Oxygen-dependent choline dehydrogenase	2.71	*ND*
*CH51_RS14770*	Betaine-aldehyde dehydrogenase	3.33	*ND*

*ND, not done.*

### Verification of Metabolic Variation in *phoU1* and *phoU2* Deletion Mutants

Based on PPI analysis, we investigated the pathways of phosphate, carbon, and pyruvate metabolism using assays of Pi, polyP, glucose, pyruvate, and ATP, respectively.

**FIGURE 3:**
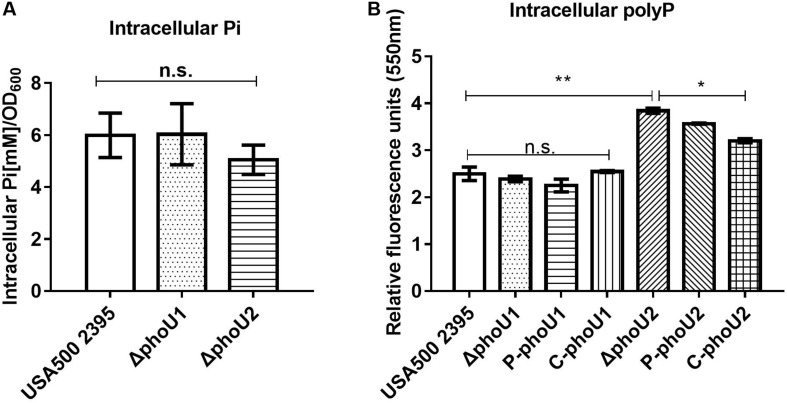
Intracellular Pi and polyP levels in Δ*phoU1* and Δ*phoU2* of *Staphylococcus aureus*. **(A)** Intracellular Pi. Bacteria were grown for 12 h; then, the cells were homogenized by OD_600_ and lysed with 0.1-mm glass-silica beads in a BeadBeater apparatus. Centrifugation to get the supernatant. The supernatant was used to measure OD_650_. Pi levels were determined according to the manufacturer’s instructions. **(B)** Intracellular polyP. Bacteria were grown for 12 h; then, the cells were homogenized by OD_600_ and incubated for 15 mins with 4’-6-diamidino-2-phenylindole. The fluorescence signal was determined at an excitation of 415 nm and an emission of 550 nm. The experiments were repeated three times, and error bars indicate the standard deviation. Δ*phoU2* exhibited significant differences (**P < 0.01) when compared with the wild-type and the complemented strains (C-*phoU2*, *P < 0.05) in intracellular polyP. The complementation of Δ*phoU1* or Δ*phoU2* mutant was abbreviated as C-*phoU1* or C-*phoU2.* The empty vectors of pCN51 transferred into the Δ*phoU1* or Δ*phoU2* mutant were named P-*phoU1* or P-*phoU2.* n.s., no significance.

PhoU homologs have been identified as phosphate-specific transport system accessory proteins. We quantified the Pi concentration of USA500 2395, Δ*phoU1*, and Δ*phoU2* strains when grown in culture for 12 h. The intracellular Pi concentration of Δ*phoU1* and Δ*phoU2* was similar to that observed in USA500 2395 (Figure 3A). The intracellular polyP level was determined using a DAPI-based fluorescence approach. The Δ*phoU2* mutant accumulated a significantly higher level of polyP (1.5-fold) than the parent strain after 12 h of growth (Figure 3B). The complemented strains of C-*phoU2* exhibited restored intracellular polyP levels.

**FIGURE 4:**
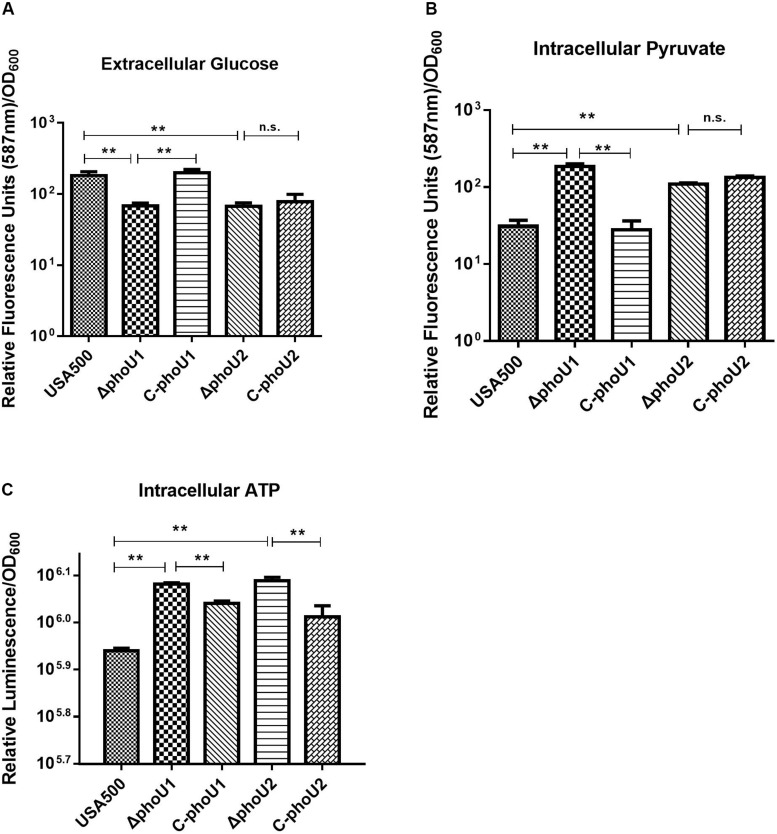
Extracellular glucose, intracellular pyruvate, and ATP in Δ*phoU1* and Δ*phoU2* of *Staphylococcus aureus*. **(A)** Extracellular glucose. Bacteria were grown for 12 h, and then the culture supernatant was harvested. Fluorescence signal was determined at an excitation of 535 nm and an emission of 587 nm. **(B)** Intracellular pyruvate. Bacteria were grown for 12 h; then, the cells were homogenized by OD_600_ and lysed with 0.1-mm glass-silica beads in a BeadBeater apparatus. Centrifugation to get the supernatant. The fluorescence signal was determined at an excitation of 535 nm and an emission of 587 nm. **(C)** Intracellular ATP. Bacteria were grown for 12 h; then, the cells were homogenized by OD_600_ and added equal to the volume of BacTiter-Glo^TM^ Reagent. Luminescence was determined. The experiments were repeated three times, and error bars indicate the standard deviation. Δ*phoU1* and Δ*phoU2* exhibited significant differences (**P < 0.01) when compared with the wild type in extracellular glucose, intracellular pyruvate, and ATP. The complemented strains of C-*phoU1* exhibited restored glucose, pyruvate, and ATP levels, and C-*phoU2* exhibited restored ATP levels.

Glucose and pyruvate levels in the mutants Δ*phoU1* and Δ*phoU2* were determined using an Abcam assay kit after growth for 12 h. Our data indicated that the extracellular glucose levels of Δ*phoU1* (68.05 ± 6.84) and Δ*phoU2* (66.86 ± 8.34) were reduced when compared to those of USA500 2395 (182.93 ± 23.17; *P* < 0.01) (Figure 4A). The intracellular pyruvate level of Δ*phoU1* (184.26 ± 15.70) and Δ*phoU2* (109.3 ± 3.55) was significantly increased when compared to the parent strain (30.99 ± 6.11) (Figure 4B). The complemented strains of C-*phoU1* exhibited restored glucose and pyruvate levels, but C-*phoU2* had no difference with the wild-type strain.

The intracellular ATP level of Δ*phoU1* and Δ*phoU2* after growth for 12 h was determined using a Promega BacTiter Glo kit. The results showed that the Δ*phoU1* and Δ*phoU2* mutant cells accumulated a significantly higher level of ATP (1.4-fold) than the parent strain (Figure 4C). The complemented strains of C-*phoU1* and C-*phoU2* exhibited restored ATP levels.

### Influence of *phoU1* and *phoU2* Deletion Mutations on Virulence

The differentially expressed genes of the Δ*phoU1* and Δ*phoU2* mutants that were related to virulence, including the type VII secretion system operon (that promotes long-term bacterial persistence) and hemolysin, were investigated by assays of invasion and survival in A549 human lung epithelial cells and by measuring the activity and the expression of α-hemolysin in culture supernatants.

**FIGURE 5:**
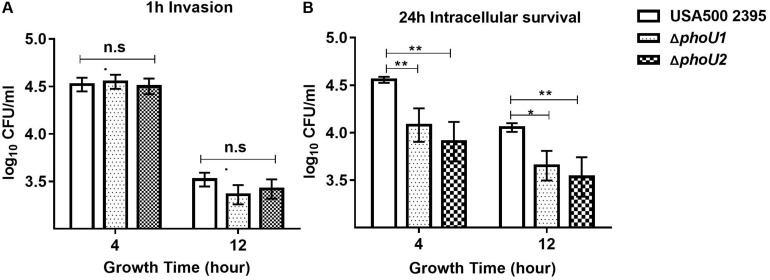
Invasion and intracellular survival of USA500 2395, Δ*phoU1*, and Δ*phoU2* in the human lung epithelial cell line A549. Bacteria grown for 4 and 12 h were used to inoculate the A549 cells at a multiplicity of infection of 10:1. **(A)** Intracellular bacteria were counted for the colony-forming units after 1 h to determine the invasion capacity and **(B)** after 24 h to assay intracellular survival. The experiments were repeated three times, and error bars indicate the standard deviation. The 4- and 12-h-culture Δ*phoU1* and Δ*phoU2* exhibited significant differences (**P < 0.01; *P < 0.05) when compared with the wild type after 24 h of intracellular survival.

For *S. aureus*, which is an important opportunistic pathogen that causes community-acquired pneumonia, assays of invasion and survival were detected using A549 human lung epithelial cells as previously described (Liang et al., 2006; Sharma et al., 2013; Mechler et al., 2015). After culturing for 4 h (log phase) or 12 h (stationary phase), wild-type, Δ*phoU1*, and Δ*phoU2* mutants were inoculated into A549 cells at a multiplicity of infection of 10:1. After incubation for 1 h, the viable bacterial count showed no significant difference between Δ*phoU1*, Δ*pho*U2, and USA500 2395, whereas after 24 h of incubation, the 4- or 12-h viable bacterial count of Δ*phoU1* (4.079 ± 0.10 log_10_)/(3.65 ± 0.09 log_10_) and Δ*phoU2* (3.90 ± 0.12 log_10_)/(3.53 ± 0.12 log_10_) showed 15–18% significant decrease compared with that observed in USA500 2395 (4.55 ± 0.18 log_10_)/(4.05 ± 0.03 log_10_) (Figure 5).

**FIGURE 6:**
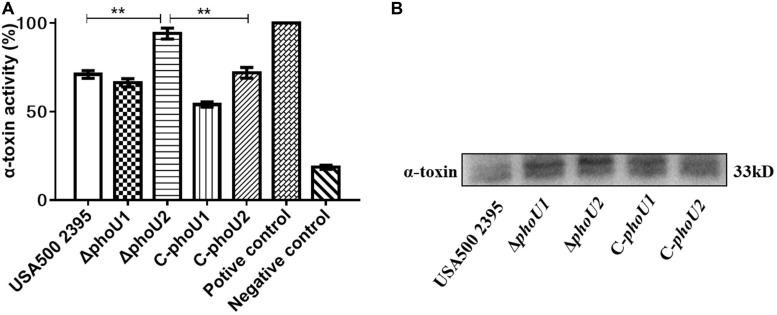
Extracellular alpha-hemolysin activity and secretion of USA500 2395, Δ*phoU1*, and Δ*phoU2*. Bacteria were grown for 12 h; then, the culture supernatant was harvested and filtered through a 0.22 μm filter. **(A)** Extracellular alpha-hemolysin activity. The supernatant was added equal to the volume of 1% of rabbit erythrocytes and then incubated at 37°C for 30 min, followed by centrifugation to obtain the supernatant. The supernatant was used to measure OD550; 0.1% triton X-100 served as the 100% hemolysis control (positive control), and 1 × phosphate-buffered saline was the negative control. **(B)** Western blot analysis of extracellular alpha-hemolysin. The experiments were repeated three times, and error bars indicate the standard deviation. Δ*phoU2* exhibited significant differences (**P < 0.01) when compared with the wild-type and the complemented strain C-*phoU2*. The complementation of Δ*phoU1* or Δ*phoU2* mutant was abbreviated as C-*phoU1* or C-*phoU2.* The empty vector of pCN51 transferred into the Δ*phoU1* or Δ*phoU2* mutant was named P-*phoU1* or P-*phoU2.*

The hemolytic activity of the *phoU* homolog mutants at 12 h (stationary phase) was determined using rabbit erythrocytes. The α-hemolysin activity of Δ*phoU2* (94.02%), was significantly enhanced compared with that of USA500 2395 (70.96%) (*P* < 0.01), whereas the α-hemolysin activity of Δ*phoU1* (66.25%) was lower than that in USA500 2395 (70.96%) (Figure 6A). Western blot analysis indicated that the α-hemolysin levels in Δ*phoU2* were significantly higher than in USA500 2395; however, no significant difference was found between the α-hemolysin level in Δ*pho*U1 and USA500 2395 (Figure 6B). Complementation of the Δ*phoU1* and Δ*phoU2* mutants restored the expression and the activity of α-hemolysin to the wild type’s level.

## Discussion

PhoU is a negative regulator of persister formation in most bacteria (Li and Zhang, 2007; Shi and Zhang, 2010). Mounting evidence has indicated the species-specific functions of PhoU homologs in bacteria; however, the impact of these proteins on bacterial growth, persister production, biofilm formation, and virulence of *S. aureus* is poorly understood. In this study, deletion of either *phoU1*or *phoU2* of *S. aureus* resulted in persister reduction in the presence of antibiotics (vancomycin or levofloxacin). The reduced persister formation in the Δ*phoU1* and Δ*phoU2* mutants is not due to a decreased intrinsic resistance to antibiotics as the MIC/MBCs of mutants show no change in antibiotic susceptibility tests. Our results suggest that both *phoU1* and *phoU2* are required for antibiotic tolerance in *S. aureus*, whereas a previous study showed that *phoU2*, but not *phoU1*, deletion resulted in persister reduction in *S. epidermidis* (Wang et al., 2017). Inactivation of *phoU* in *E. coli* and *P. aeruginosa* has been shown to reduce antibiotic susceptibility and persister formation (Li and Zhang, 2007; de Almeida et al., 2015). Deletion of *phoY2* in *M. tuberculosis* H37Rv increased the susceptibility to pyrazinamide and rifampicin and reduced persister formation (Shi and Zhang, 2010). However, single deletions of *phoY1* and *phoY2* mutants of the *M. tuberculosis* Erdman strain showed no impact on drug susceptibility and persister formation (Namugenyi et al., 2017). By comparison, the double-deletion *phoY1* and *phoY2* mutants in the *M. tuberculosis* Erdman strain exhibited a decrease in persister formation and specifically enhanced the susceptibility to rifampicin, but not to other antimycobacterial drugs. Growth curve and survival assays were both measured in the presence of the cell wall-disrupting detergent sodium dodecyl sulfate and the reactive oxygen hydrogen peroxide (H_2_O_2_). Our results suggest that both *phoU1* and *phoU2* are required for resistance to SDS and H_2_O_2_ in *S. aureus*. Inactivation of *phoU* in *E. coli*, *P. aeruginosa*, and *S. epidermidis* has been shown to reduce tolerance to H_2_O_2_. This suggests that the functions and the regulatory mechanisms of *phoU* homologs in relation to antimicrobial susceptibility and persister formation are distinct in different bacteria.

Persisters are often slow growing or non-growing with reduced metabolism (Maisonneuve and Gerdes, 2014; Prax and Bertram, 2014; Cabral et al., 2018; Kaldalu and Tenson, 2019). Deletion of *phoU* in *E. coli* and *P. aeruginosa* slowed the growth rates but increased the metabolism (Li and Zhang, 2007; de Almeida et al., 2015). Single Δ*phoY1* or Δ*phoY2* mutants of *M. tuberculosis* had no significant effects on bacterial growth but decreased the viable bacteria count of Δ*phoY1*Δ*phoY2* double mutant at the stationary phase (Wang et al., 2013; Namugenyi et al., 2017). The *S. epidermidis phoU2* deletion resulted in growth retardation, whereas the present study demonstrated that the growth curve and the viable bacterial count of *S. aureus* Δ*phoU1* and Δ*phoU2* were the same as that of the parent strain, which were confirmed by silenced *phoU1* or *phoU2* in USA500 2395, USA300, and SA113 strains (Supplementary Figure S2).

The mechanisms of persister formation are complex. Intracellular polyP level is associated with antibiotic tolerance and persister formation. PolyP accumulation has been associated with increased persister frequency in both *E. coli* and *M. tuberculosis* (Thayil et al., 2011; Singh et al., 2013; Chuang et al., 2015; Germain et al., 2015). Differently, the enhanced polyP accumulation, resulting from the inactivation or the deletion of *phoU* in *M. tuberculosis*, *E. coli*, *P. aeruginosa*, or *S. epidermidis*, was accompanied by a decreased sensitivity to antibiotics and persister generation (Morohoshi et al., 2002; Li and Zhang, 2007; de Almeida et al., 2015; Namugenyi et al., 2017; Wang et al., 2017). These data were consistent with our current study in terms of intracellular polyP accumulation and persister generation reduction in the Δ*phoU1* and the Δ*phoU2* strains of *S. aureus*.

The bacterial metabolic state is a major determinant of persister. This view can be used to explain the bacteria in the biofilm. Cells in the periphery of the biofilm, where metabolic activity is highest, indicate that antibiotic efficacy is highest. The cells in the center of the biofilm exhibit a marked dormancy and low antibiotic efficiency (Walters et al., 2003). Another example is starvation stress, which induces changes in the expression of metabolic pathways such as energy metabolism, amino acid metabolism, and lipid metabolism, while long-term starvation of *M. tuberculosis* reduces susceptibility to rifampicin, isoniazid, and metronidazole (Betts et al., 2002). Most antibiotics, such as quinolones, aminoglycosides, and β-lactams, kill bacteria by corrupting targets which are energy dependent. Quinolones inhibit the ligase activity of gyrase and topoisomerase and release DNA with single- and double-strand breaks that lead to cell death (Hooper, 2001). Aminoglycoside acts by producing toxic misfolded peptides (Davis, 1987). Beta-lactams exert their antibacterial effect by irreversibly binding to the Ser residue of the penicillin-binding protein active site, forcing a futile cycle of peptidoglycan synthesis (Cho et al., 2014). Those targets require ATP to function. Recent studies showed that lowering intracellular ATP with arsenate treatment could increase the level of persister formation in *E. coli* and *S. aureus* under fluoroquinolones (Conlon et al., 2016; Shan et al., 2017). Persister formation is associated with ATP depletion in *E. coli* and *S. aureus* (Conlon et al., 2016; Shan et al., 2017). ATP can be produced by multiple cellular pathways, including oxidative phosphorylation, ATP synthase, and polyP, which can be converted to ATP (Resnick and Zehnder, 2000; Bonora et al., 2012). The change of metabolism pathways may be an association with various factors, and more comprehensive considerations are required. Hence, the results of metabolic products would become more convincing. Our result showed that the *S. aureus* Δ*phoU1* or Δ*phoU2* mutant decreased the level of persister formation by hyperactive carbon metabolism (decreased extracellular glucose and intracellular pyruvate) accompanied by increasing intracellular ATP. In the *S. aureus* Δ*phoU1* mutant, the transcriptome analysis revealed that 26 genes up-regulated by twofold and three genes down-regulated by two- to threefold, identified to be involved in carbon, pyruvate, and glycolysis metabolism, consistent with the hyperactive carbon metabolism and increasing intracellular ATP. In the *S. aureus* Δ*phoU2* mutant, our analysis showed that 13 genes up-regulated by three- to 24-fold of transported phosphate. The increasing intracellular polyP was consistent with the high intracellular ATP content in the Δ*phoU2* mutant. However, three genes (D-lactate dehydrogenase, pyruvate formate lyase, and alcohol dehydrogenase) were up-regulated by threefold and 10 genes were down-regulated by twofold in carbon, pyruvate, and glycolysis metabolism. Lactate dehydrogenase (catalyzes the interconversion of pyruvate and lactate), pyruvate formate lyase (regulates the formation of acetyl-CoA, which is important in the production of energy), and alcohol dehydrogenase (converts pyruvate to acetaldehyde and involved in the production of ATP) play an important part in carbon, pyruvate, and glycolysis metabolism (Garvie, 1980; Lamed and Zeikus, 1981; Hasona et al., 2004). It is possible that the result of the Δ*phoU2* mutant presented decreased extracellular glucose and intracellular pyruvate (Wang et al., 2017). Those results suggest that, in *S. aureus* Δ*phoU1* or Δ*phoU2*, decreased tolerance to antibiotics or stresses of SDS and H_2_O_2_ may be associated with higher levels of carbon metabolism and intracellular ATP. Complementary strains C-*phoU1*, C-*phoU2*, and C-*phoU1phoU2* restored the majority of phenotypes, and only the C-*phoU2* strains failed to restore the phenotypes of glucose and pyruvate, indicating that the results were not due to a secondary mutation elsewhere on the chromosome. The reason may be due to the PhoU, a global metabolic repressor. Regulation has a dual function which is reflected in the induced response to phosphate limitation but also inhibited when Pi is in excess (Gardner et al., 2014; Lubin et al., 2015; diCenzo et al., 2017). The expression of the *phoU1* or the *phoU2* in the complementary strains C-*phoU1* and C-*phoU2* were around three- or fivefold higher than that in the wild-type strain. Excessive *phoU2* expression may cause diminished compensation function in glucose and pyruvate phenotypes. This may not be a unique reason as bacteria have a complex metabolism regulatory system and thus need further study.

The intracellular invasion and the survival rates of *S. aureus* are crucial for the pathogenicity of chronic infection (Rollin et al., 2017; Tan et al., 2019). Several virulence factors play a critical role in the pathogenic procession. We found that either *phoU1* or *phoU2* is required for the intracellular survival of *S. aureus* in human lung epithelial A549 cells. In the method of invasion and intracellular survival assays, gentamicin (10 μg/ml) was added in the culture medium to restrict the extracellular growth of bacteria when *S. aureus* and A549 cells were co-incubated. Gentamicin and erythromycin may have an antagonizing effect (Penn et al., 1982), so we could not perform the invasion and the intracellular survival assays on the complemented strains of C-*phoU1* and C-*phoU2*. However, a previous report, as a piece of supporting evidence, showed that a single-point mutation in *pitA* (downstream of *phoU2*) resulted in a decrease in the intracellular survival of *S. aureus* in human epithelial cells (Mechler et al., 2015). The deletion of *phoU1* or *phoU2* in *S. aureus* resulted in the down-regulation of multiple virulence systems, including the type VII secretion system, serine protease, and leucocidin, which could explain the decreased survival of the *phoU1* and the *phoU2* mutants.

The α-hemolysin is a major virulence factor in *S. aureus* infections (Kebaier et al., 2012; Vandenesch et al., 2012). We observed that the α-hemolysin activity and its expression were increased in Δ*phoU2*, while there was no effect noted in Δ*phoU1*. This suggests that *phoU2*, but not *phoU1*, is a negative regulator of α-hemolysin. However, the transcription of α-hemolysin in Δ*phoU2* was reduced after 12 h of growth when assessed by RNA-Seq and RT-PCR.

The virulence regulation mechanisms of *S. aureus* are very complex (Bronner et al., 2004). The virulence regulatory systems include two-component systems, including AgrAC, SaeRS, ArlRS, etc., and the global regulator, including cytoplasmic SarA-family, CodY, Rot, etc. (Haag and Bagnoli, 2017). Among them, AgrAC, SarA, and SaeRS are positive regulators, whereas CodY and Rot are negative regulators of virulence genes (Pragman and Schlievert, 2004). The deletion of *phoU1* resulted in a decreasing expression of *sarA* and *saeS* and increasing expressions of *codY*. The deletion of *phoU2* resulted in decreasing expressions of *sarA* family (*sarA*, *sarR*, and *sarZ*), *codY*, and *rot* and an increasing expression of *phoRP*, the function of which is unknown. Therefore, the regulatory mechanisms of *phoU1* and *phoU2* in relation to virulence require further study.

*S. aureus* biofilm formation also plays an important role in persister production during chronic infections (Archer et al., 2011; Conlon, 2014). *S. aureus* SA113 is a biofilm positive strain characterized by *rsbU*, *tcaR*, and *agr* mutants (Herbert et al., 2010). Our results showed that silencing *phoU2*, but not *phoU1*, can reduce biofilm formation in the *S. aureus* SA113 strain (Supplementary Figure S3). This is consistent with the previous finding in *S. epidermidis* that PhoU2, but not PhoU1, is an important regulator of biofilm formation.

In summary, both PhoU1 and PhoU2 of *S. aureus* regulate persister generation and bacterial virulence. The deletion of *phoU1* or *phoU2* of *S. aureus* resulted in a decrease in intracellular bacterial survival rate in human epithelial cells by down-regulation of multiple virulence factors, including the type VII secretion system, serine protease, and leucocidin. In a follow-up work, the double mutant of Δ*phoU1*Δ*phoU2* was constructed, and the metabolic variation was similar to the single mutant in extracellular glucose, intracellular pyruvate, ATP, and intracellular polyP levels (Supplementary Figure S5). The results in *S. aureus* are different from what we have previously found in *S. epidermidis*, where only PhoU2 regulates biofilm and persister formation. In *S. aureus*, transcriptome analysis revealed that 573 or 285 genes were differentially expressed in the Δ*phoU1* or the Δ*phoU2* mutant *vs* the wild type. In *S. epidermidis*, deletion of *phoU1* just led to 92 differentially expressed genes, while deletion of *phoU2* could result in 945 differentially expressed genes (Wang et al., 2017). In *S. aureus*, both *phoU1* and *phoU2* regulate virulence by the global regulator (*sarA*, *rot*, and *codY*) and persister generation by the hyperactive carbon metabolism accompanied by increasing intracellular ATP. In the *S. epidermidis* Δ*phoU2* mutant, the reduction of biofilm and persister can be explained by the down-regulated expression of several important genes involved in growth (including *yycFG*, *rsuU*, *pflA*, and *nrdD*) and increased ATP by up-regulated ATP synthase (Dubrac et al., 2008; Wang et al., 2017). In the previous studies, we found that the regulatory mechanisms of some global regulators (SaeRS, YycFG, SrrAB, and ArlRS) have different roles between *S. aureus* and *S. epidermidis* (Lou et al., 2011; Wu et al., 2012, 2015; Xu et al., 2017). These observations hint that different mechanisms occur in the regulation of *S. aureus* and *S. epidermidis*, even though they belong to the staphylococcus. Therefore, the different regulation mechanisms of PhoU homologs in *S. aureus* and *S. epidermidis* warrant further investigation in the future.

## Data Availability Statement

All datasets generated for this study are included in the article/Supplementary Material.

## Author Contributions

DQ, YZ, and ZY designed the research. YS, XW, and ZC participated in most of the experiments. ZLy, ZLi, JZ, YW, and QD analyzed the data. YS drafted the manuscript. DQ, YZ, and ZY revised the manuscript.

## Conflict of Interest

The authors declare that the research was conducted in the absence of any commercial or financial relationships that could be construed as a potential conflict of interest.

## Footnotes

1 http://www.genome.jp/kegg/pathway.html

## References

[B1] Archer N. K. Mazaitis M. J. Costerton J. W. Leid J. G. Powers M. E. Shirtliff M. E. (2011). *Staphylococcus aureus* biofilms: properties, regulation, and roles in human disease. *Virulence* 2 445–459. 10.4161/viru.2.5.17724 21921685 PMC3322633

[B2] Aschar-Sobbi R. Abramov A. Y. Diao C. Kargacin M. E. Kargacin G. J. French R. J. (2008). High sensitivity, quantitative measurements of polyphosphate using a new DAPI-based approach. *J. Fluoresc.* 18 859–866. 10.1007/s10895-008-0315-4 18210191

[B3] Bae T. Schneewind O. (2006). Allelic replacement in *Staphylococcus aureus* with inducible counter-selection. *Plasmid* 55 58–63. 10.1016/j.plasmid.2005.05.005 16051359

[B4] Bernheimer A. W. Avigad L. S. Grushoff P. (1968). Lytic effects of staphylococcal alpha-toxin and delta-hemolysin. *J. Bacteriol.* 96 487–491. 10.1128/jb.96.2.487-491.1968 4970650 PMC252322

[B5] Betts J. C. Lukey P. T. Robb L. C. McAdam R. A. Duncan K. (2002). Evaluation of a nutrient starvation model of *Mycobacterium tuberculosis* persistence by gene and protein expression profiling. *Mol. Microbiol.* 43 717–731. 10.1046/j.1365-2958.2002.02779.x 11929527

[B6] Bonora M. Patergnani S. Rimessi A. De Marchi E. Suski J. M. Bononi A. (2012). ATP synthesis and storage. *Purinergic Signal.* 8 343–357. 10.1007/s11302-012-9305-8 22528680 PMC3360099

[B7] Bonora M. Wieckowsk M. R. Chinopoulos C. Kepp O. Kroemer G. Galluzzi L. (2015). Molecular mechanisms of cell death: central implication of ATP synthase in mitochondrial permeability transition. *Oncogene* 34:1608 10.1038/onc.2014.462 25790189

[B8] Bronner S. Monteil H. Prévost G. (2004). Regulation of virulence determinants in *Staphylococcus aureus*: complexity and applications. *FEMS Microbiol. Rev.* 28 183–200. 10.1016/j.femsre.2003.09.003 15109784

[B9] Cabral D. J. Wurster J. I. Belenky P. (2018). Antibiotic persistence as a metabolic adaptation: stress, metabolism, the host, and new directions. *Pharmaceuticals* 11;14. 10.3390/ph11010014 PMC5874710 29389876

[B10] Cho H. Uehara T. Bernhardt T. G. (2014). Beta-lactam antibiotics induce a lethal malfunctioning of the bacterial cell wall synthesis machinery. *Cell* 159 1300–1311. 10.1016/j.cell.2014.11.017 25480295 PMC4258230

[B11] Chuang Y.-M. Bandyopadhyay N. Rifat D. Rubin H. Bader J. S. Karakousis P. C. (2015). Deficiency of the novel exopolyphosphatase Rv1026/PPX2 leads to metabolic downshift and altered cell wall permeability in *Mycobacterium tuberculosis*. *mBio* 6:e02428-14 10.1128/mBio.02428-14 PMC4453511 25784702

[B12] Conlon B. P. (2014). *Staphylococcus aureus* chronic and relapsing infections: evidence of a role for persister cells: an investigation of persister cells, their formation and their role in *S. aureus* disease. *Bioessays* 36 991–996. 10.1002/bies.201400080 25100240

[B13] Conlon B. P. Rowe S. E. Gandt A. B. Nuxoll A. S. Donegan N. P. Zalis E. A. (2016). Persister formation in *Staphylococcus aureus* is associated with ATP depletion. *Nat. Microbiol.* 1:16051 10.1038/nmicrobiol.2016.51 PMC4932909 27572649

[B14] Davis B. D. (1987). Mechanism of bactericidal action of aminoglycosides. *Microbiol. Rev.* 51 341–350. 10.1128/mmbr.51.3.341-350.1987 3312985 PMC373115

[B15] de Almeida L. G. Ortiz J. H. Schneider R. P. Spira B. (2015). phoU inactivation in *Pseudomonas aeruginosa* enhances accumulation of ppGpp and polyphosphate. *Appl. Environ. Microbiol.* 81 3006–3015. 10.1128/AEM.04168-14 25710363 PMC4393453

[B16] diCenzo G. C. Sharthiya H. Nanda A. Zamani M. Finan T. M. (2017). PhoU allows rapid adaptation to high phosphate concentrations by modulating PstSCAB transport rate in *Sinorhizobium meliloti*. *J. Bacteriol.* 199, e00143–00117. 10.1128/jb.00143-17 28416708 PMC5573078

[B17] Dinges M. M. Orwin P. M. Schlievert P. M. (2000). Exotoxins of *Staphylococcus aureus*. *Clin.Microbiol. Rev.* 13;16 10.1128/CMR.13.1.16 PMC88931 10627489

[B18] Dubrac S. Bisicchia P. Devine K. M. Msadek T. (2008). A matter of life and death: cell wall homeostasis and the WalKR (YycGF) essential signal transduction pathway. *Mol. Microbiol.* 70 1307–1322. 10.1111/j.1365-2958.2008.06483.x 19019149

[B19] El-Halfawy O. M. Valvano M. A. (2015). Antimicrobial heteroresistance: an emerging field in need of clarity. *Clin. Microbiol. Rev.* 28 191–207. 10.1128/CMR.00058-14 25567227 PMC4284305

[B20] Fisher R. A. Gollan B. Helaine S. (2017). Persistent bacterial infections and persister cells. *Nat. Rev. Microbiol.* 15:453 10.1038/nrmicro.2017.42 28529326

[B21] Fraunholz M. Sinha B. (2012). Intracellular *Staphylococcus aureus*: live-in and let die. *Front. Cell. Infect. Microbiol.* 2:43 10.3389/fcimb.2012.00043 PMC3417557 22919634

[B22] Gardner S. G. Johns K. D. Tanner R. McCleary W. R. (2014). The PhoU protein from Escherichia coli interacts with *PhoR*, *PstB*, and metals to form a phosphate-signaling complex at the membrane. *J. Bacteriol.* 196, 1741–1752. 10.1128/JB.00029-14 24563032 PMC3993317

[B23] Garvie E. I. (1980). Bacterial lactate dehydrogenases. *Microbiol. Rev.* 44 106–139. 10.1128/mmbr.44.1.106-139.1980 6997721 PMC373236

[B24] Germain E. Roghanian M. Gerdes K. Maisonneuve E. (2015). Stochastic induction of persister cells by HipA through (p)ppGpp-mediated activation of mRNA endonucleases. *Proc. Natl. Acad. Sci. U.S.A.* 112 5171–5176. 10.1073/pnas.1423536112 25848049 PMC4413331

[B25] Grassi L. Di Luca M. Maisetta G. Rinaldi A. C. Esin S. Trampuz A. (2017). Generation of persister cells of *Pseudomonas aeruginosa* and *Staphylococcus aureus* by chemical treatment and evaluation of their susceptibility to membrane-targeting agents. *Front. Microbiol.* 8:1917 10.3389/fmicb.2017.01917 PMC5632672 29046671

[B26] Haag A. F. Bagnoli F. (2017). The role of two-component signal transduction systems in *Staphylococcus aureus* virulence regulation. *Curr. Top. Microbiol. Immunol.* 409 145–198. 10.1007/82_2015_5019 26728068

[B27] Hasona A. Kim Y. Healy F. G. Ingram L. O. Shanmugam K. T. (2004). Pyruvate formate lyase and acetate kinase are essential for anaerobic growth of *Escherichia coli* on xylose. *J. Bacteriol.* 186 7593–7600. 10.1128/JB.186.22.7593-7600.2004 15516572 PMC524897

[B28] Helle L. Kull M. Mayer S. Marincola G. Zelder M. E. Goerke C. (2011). Vectors for improved Tet repressor-dependent gradual gene induction or silencing in *Staphylococcus aureus*. *Microbiology* 157(Pt 12), 3314–3323. 10.1099/mic.0.052548-0 21921101

[B29] Herbert S. Ziebandt A. K. Ohlsen K. Schafer T. Hecker M. Albrecht D. (2010). Repair of global regulators in *Staphylococcus aureus* 8325 and comparative analysis with other clinical isolates. *Infect. Immun.* 78 2877–2889. 10.1128/IAI.00088-10 20212089 PMC2876537

[B30] Hooper D. C. (2001). Mechanisms of action of antimicrobials: focus on fluoroquinolones. *Clin. Infect. Dis.* 32(Suppl. 1), S9–S15. 10.1086/319370 11249823

[B31] Kaldalu N. Tenson T. (2019). Slow growth causes bacterial persistence. *Sci. Signal.* 12:eaay1167 10.1126/scisignal.aay1167 31363066

[B32] Kebaier C. Chamberland R. R. Allen I. C. Gao X. Broglie P. M. Hall J. D. (2012). *Staphylococcus aureus* α-hemolysin mediates virulence in a murine model of severe pneumonia through activation of the NLRP3 inflammasome. *J. Infect. Dis.* 205 807–817. 10.1093/infdis/jir846 22279123 PMC3274379

[B33] Lamed R. J. Zeikus J. G. (1981). Novel NADP-linked alcohol–aldehyde/ketone oxidoreductase in thermophilic ethanologenic bacteria. *Biochem. J.* 195 183–190. 10.1042/bj1950183 7030321 PMC1162870

[B34] Li Y. Zhang Y. (2007). PhoU is a persistence switch involved in persister formation and tolerance to multiple antibiotics and stresses in *Escherichia coli*. *Antimicrob. Agents Chemother.* 51 2092–2099. 10.1128/AAC.00052-07 17420206 PMC1891003

[B35] Liang X. Yee S. W. Chien H. C. Chen E. C. Luo Q. Zou L. (2018). Organic cation transporter 1 (OCT1) modulates multiple cardiometabolic traits through effects on hepatic thiamine content. *PLoS Biol.* 16:e2002907 10.1371/journal.pbio.2002907 PMC5919692 29659562

[B36] Liang X. Yu C. Sun J. Liu H. Landwehr C. Holmes D. (2006). Inactivation of a two-component signal transduction system, SaeRS, eliminates adherence and attenuates virulence of *Staphylococcus aureus*. *Infect. Immun.* 74 4655–4665. 10.1128/IAI.00322-06 16861653 PMC1539584

[B37] Liu J. Lou Y. Yokota H. Adams P. D. Kim R. Kim S. H. (2005). Crystal structure of a PhoU protein homologue: a new class of metalloprotein containing multinuclear iron clusters. *J. Biol. Chem.* 280 15960–15966. 10.1074/jbc.M414117200 15716271

[B38] Lou Q. Zhu T. Hu J. Ben H. Yang J. Yu F. (2011). Role of the SaeRS two-component regulatory system in *Staphylococcus epidermidis* autolysis and biofilm formation. *BMC Microbiol.* 11:146 10.1186/1471-2180-11-146 PMC3224141 21702925

[B39] Lubin E. A. Henry J. T. Fiebig A. Crosson S. Laub M. T. (2015). Identification of the PhoB regulon and role of phou in the phosphate starvation response of *Caulobacter crescentus*. *J. Bacteriol.* 198, 187–200. 10.1128/JB.00658-15 26483520 PMC4686198

[B40] Maisonneuve E. Gerdes K. (2014). Molecular mechanisms underlying bacterial persisters. *Cell* 157 539–548. 10.1016/j.cell.2014.02.050 24766804

[B41] Mechler L. Bonetti E.-J. Reichert S. Flötenmeyer M. Schrenzel J. Bertram R. (2016). Daptomycin tolerance in the *Staphylococcus aureus* pitA6 mutant is due to upregulation of the dlt operon. *Antimicrob. Agents Chemother.* 60 2684–2691. 10.1128/AAC.03022-15 26883712 PMC4862447

[B42] Mechler L. Herbig A. Paprotka K. Fraunholz M. Nieselt K. Bertram R. (2015). A novel point mutation promotes growth phase-dependent daptomycin tolerance in *Staphylococcus aureus*. *Antimicrob. Agents Chemother.* 59 5366–5376. 10.1128/aac.00643-15 26100694 PMC4538524

[B43] Moormeier D. E. Bayles K. W. (2017). *Staphylococcus aureus* biofilm: a complex developmental organism. *Mol. Microbiol.* 104 365–376. 10.1111/mmi.13634 28142193 PMC5397344

[B44] Morohoshi T. Maruo T. Shirai Y. Kato J. Ikeda T. Takiguchi N. (2002). Accumulation of inorganic polyphosphate in mutants of *Escherichia coli* and Synechocystis sp. Strain PCC6803. *Appl. Environ. Microbiol.* 68:4107 10.1128/AEM.68.8.4107-4110.2002 PMC124021 12147514

[B45] Namugenyi S. B. Aagesen A. M. Elliott S. R. Tischler A. D. (2017). *Mycobacterium tuberculosis* PhoY proteins promote persister formation by mediating pst/SenX3-RegX3 phosphate sensing. *mBio* 8:e00494-17 10.1128/mBio.00494-17 PMC5513712 28698272

[B46] Otto M. (2010). *Staphylococcus* colonization of the skin and antimicrobial peptides. *Expert Rev. Dermatol.* 5 183–195. 10.1586/edm.10.6 20473345 PMC2867359

[B47] Otto M. (2014). *Staphylococcus aureus* toxins. *Curr. Opin. Microbiol.* 17 32–37. 10.1016/j.mib.2013.11.004 24581690 PMC3942668

[B48] Overton I. M. Graham S. Gould K. A. Hinds J. Botting C. H. Shirran S. (2011). Global network analysis of drug tolerance, mode of action and virulence in methicillin-resistant *S. aureus*. *BMC Syst. Biol.* 5:68 10.1186/1752-0509-5-68 PMC3123200 21569391

[B49] Penn R. L. Ward T. T. Steigbigel R. T. (1982). Effects of erythromycin in combination with penicillin, ampicillin, or gentamicin on the growth of *Listeria monocytogenes*. *Antimicrob. Agents Chemother.* 22 289–294. 10.1128/aac.22.2.289 6821458 PMC183727

[B50] Pragman A. A. Schlievert P. M. (2004). Virulence regulation in *Staphylococcus aureus*: the need for in vivo analysis of virulence factor regulation. *Pathog. Dis.* 42 147–154. 10.1016/j.femsim.2004.05.005 15364098

[B51] Prax M. Bertram R. (2014). Metabolic aspects of bacterial persisters. *Front. Cell. Infect. Microbiol.* 4:148 10.3389/fcimb.2014.00148 PMC4205924 25374846

[B52] Resnick S. M. Zehnder A. J. (2000). In vitro ATP regeneration from polyphosphate and AMP by polyphosphate:AMP phosphotransferase and adenylate kinase from *Acinetobacter johnsonii* 210A. *Appl. Environ. Microbiol.* 66 2045–2051. 10.1128/aem.66.5.2045-2051.2000 10788379 PMC101452

[B53] Rollin G. Tan X. Tros F. Dupuis M. Nassif X. Charbit A. (2017). Intracellular survival of *Staphylococcus aureus* in endothelial cells: a matter of growth or persistence. *Front. Microbiol.* 8:1354 10.3389/fmicb.2017.01354 PMC5515828 28769913

[B54] Schnack L. Sohrabi Y. Lagache S. M. M. Kahles F. Bruemmer D. Waltenberger J. (2019). Mechanisms of trained innate immunity in oxLDL primed human coronary smooth muscle cells. *Front. Immunol.* 10:13 10.3389/fimmu.2019.00013 PMC6351498 30728822

[B55] Shan Y. Brown Gandt A. Rowe S. E. Deisinger J. P. Conlon B. P. Lewis K. (2017). ATP-dependent persister formation in *Escherichia coli*. *mBio* 8:e02267-16 10.1128/mBio.02267-16 PMC5296605 28174313

[B56] Sharma P. Lata H. Arya D. K. Kashyap A. K. Kumar H. Dua M. (2013). Role of pilus proteins in adherence and invasion of *Streptococcus agalactiae* to the lung and cervical epithelial cells. *J. Biol. Chem.* 288 4023–4034. 10.1074/jbc.M112.425728 23209289 PMC3567654

[B57] Sharma-Kuinkel B. K. Ahn S. H. Rude T. H. Zhang Y. Tong S. Y. Ruffin F. (2012). Presence of genes encoding panton-valentine leukocidin is not the primary determinant of outcome in patients with hospital-acquired pneumonia due to *Staphylococcus aureus*. *J. Clin. Microbiol.* 50 848–856. 10.1128/jcm.06219-11 22205797 PMC3295120

[B58] Shi W. Zhang Y. (2010). PhoY2 but not PhoY1 is the PhoU homologue involved in persisters in *Mycobacterium tuberculosis*. *J. Antimicrob. Chemother.* 65 1237–1242. 10.1093/jac/dkq103 20360062 PMC2868530

[B59] Singh R. Singh M. Arora G. Kumar S. Tiwari P. Kidwai S. (2013). Polyphosphate deficiency in *Mycobacterium tuberculosis* is associated with enhanced drug susceptibility and impaired growth in guinea pigs. *J. Bacteriol.* 195:2839 10.1128/JB.00038-13 PMC3697247 23585537

[B60] Tan X. Coureuil M. Ramond E. Euphrasie D. Dupuis M. Tros F. (2019). Chronic *Staphylococcus aureus* lung infection correlates with proteogenomic and metabolic adaptations leading to an increased intracellular persistence. *Clin. Infect. Dis.* 69 1937–1945. 10.1093/cid/ciz106 30753350

[B61] Thayil S. M. Morrison N. Schechter N. Rubin H. Karakousis P. C. (2011). The role of the novel exopolyphosphatase MT0516 in *Mycobacterium tuberculosis* drug tolerance and persistence. *PLoS One* 6:e28076 10.1371/journal.pone.0028076 PMC3221697 22132215

[B62] Tong S. Y. C. Davis J. S. Eichenberger E. Holland T. L. Fowler V. G. Jr. (2015). *Staphylococcus aureus* infections: epidemiology, pathophysiology, clinical manifestations, and management. *Clin. Microbiol. Rev.* 28 603–661. 10.1128/CMR.00134-14 26016486 PMC4451395

[B63] Vandenesch F. Lina G. Henry T. (2012). *Staphylococcus aureus* hemolysins, bi-component leukocidins, and cytolytic peptides: a redundant arsenal of membrane-damaging virulence factors? *Front. Cell. Infect. Microbiol.* 2:12 10.3389/fcimb.2012.00012 PMC3417661 22919604

[B64] Walters M. C. III Roe F. Bugnicourt A. Franklin M. J. Stewart P. S. (2003). Contributions of antibiotic penetration, oxygen limitation, and low metabolic activity to tolerance of *Pseudomonas aeruginosa* biofilms to ciprofloxacin and tobramycin. *Antimicrob. Agents Chemother.* 47 317–323. 10.1128/aac.47.1.317-323.2003 12499208 PMC148957

[B65] Wang C. Mao Y. Yu J. Zhu L. Li M. Wang D. (2013). PhoY2 of mycobacteria is required for metabolic homeostasis and stress response. *J. Bacteriol.* 195 243–252. 10.1128/jb.01556-12 23123909 PMC3553840

[B66] Wang X. Han H. Lv Z. Lin Z. Shang Y. Xu T. (2017). PhoU2 but Not PhoU1 as an important regulator of biofilm formation and tolerance to multiple stresses by participating in various fundamental metabolic processes in *Staphylococcus epidermidis*. *J. Bacteriol.* 199:e00219-17 10.1128/jb.00219-17 PMC5686610 28947672

[B67] Wilmaerts D. Windels E. M. Verstraeten N. Michiels J. (2019). General mechanisms leading to persister formation and awakening. *Trends Genet.* 35 401–411. 10.1016/j.tig.2019.03.007 31036343

[B68] Wu Y. Wang J. Xu T. Liu J. Yu W. Lou Q. (2012). The two-component signal transduction system ArlRS regulates *Staphylococcus epidermidis* biofilm formation in an ica-dependent manner. *PLoS One* 7:e40041 10.1371/journal.pone.0040041 PMC3407220 22848368

[B69] Wu Y. Wu Y. Zhu T. Han H. Liu H. Xu T. (2015). *Staphylococcus epidermidis* SrrAB regulates bacterial growth and biofilm formation differently under oxic and microaerobic conditions. *J. Bacteriol.* 197 459–476. 10.1128/jb.02231-14 25404696 PMC4285975

[B70] Xu T. Wu Y. Lin Z. Bertram R. Gotz F. Zhang Y. (2017). Identification of genes controlled by the essential YycFG two-component system reveals a role for biofilm modulation in *Staphylococcus epidermidis*. *Front. Microbiol.* 8:724 10.3389/fmicb.2017.00724 PMC5405149 28491057

