# Supplementary material for: Staphylococcus aureus PhoU Homologs Regulate Persister Formation and Virulence
Source: Front Microbiol. 2020 May 26;11:865. doi: 10.3389/fmicb.2020.00865 (PMC7326077; doi:10.3389/fmicb.2020.00865)
Supplement: Supplementary file 1 [file Data_Sheet_1.pdf]

Supplementary Material

Supplementary Figures

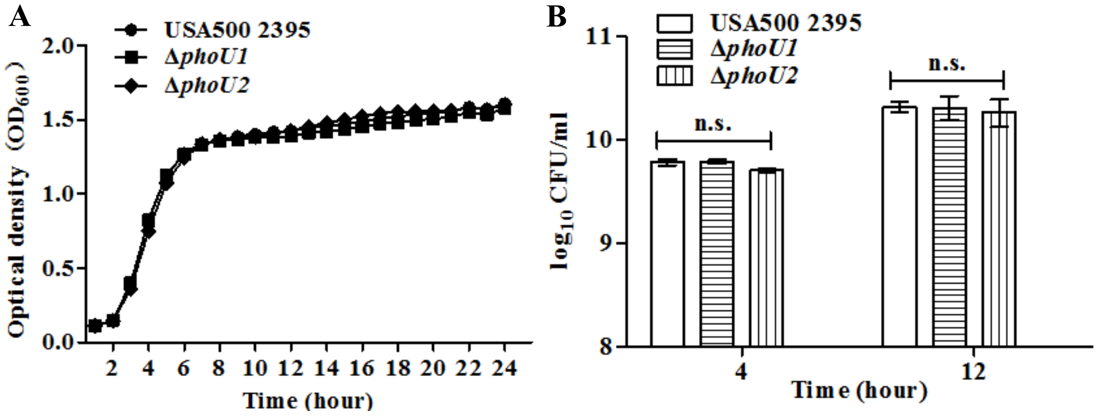

Supplementary Figure 1. Impact of *phoU1* or *phoU2* deletion on the growth of *S. aureus*. Overnight cultures of the Δ*phoU1*, Δ*phoU2*, and USA500 2395 strains were diluted 1:200 into TSB. **(A)** Bacterial growth curve. Bacterial were grown in TSB at 37 °C with shaking at 220 rpm and monitored by measuring the OD_{600} at indicated time points, until 24 h. **(B)** Viable bacteria count. Bacteria in serial dilutions were plated on TSB agar plates at 4 h and 24 h, and CFU were counts. The experiments were repeated three times, and error bars indicate the standard deviation. n.s., no significance.

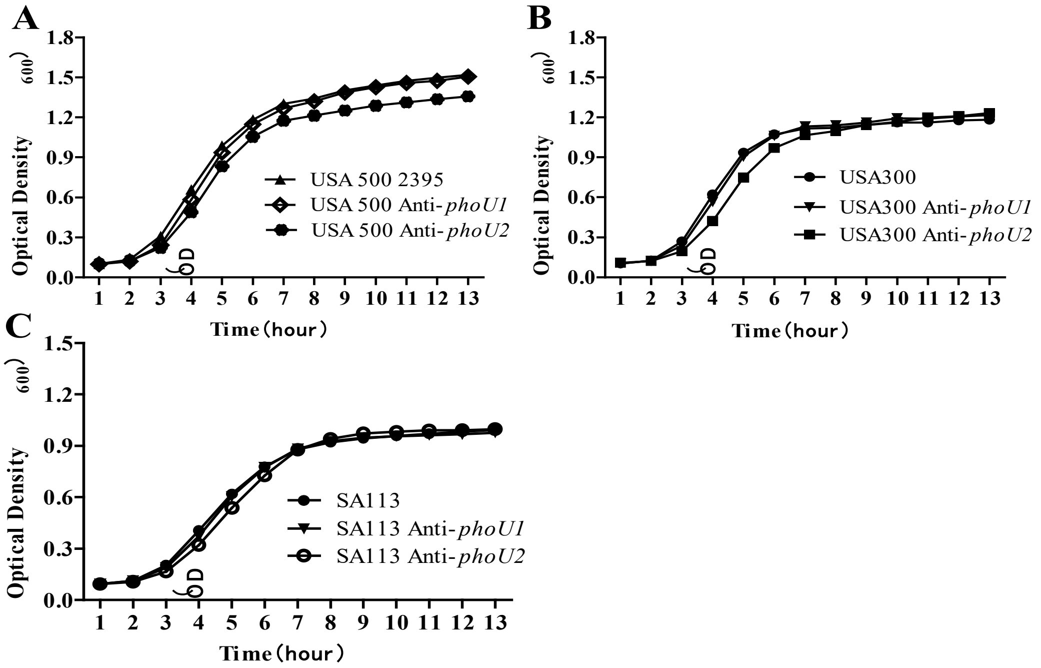

Supplementary Figure 2. Impact of *phoU1* or *phoU2* silencing on the growth of diferent *S. aureus*. Overnight cultures (12 h) of USA500 2395, USA300 and SA113 were diluted 1:200 into TSB containing 125 ng/ml anhydrotetracycline (ATc). Bacteria were grown in TSB at 37 °C with with shaking at 220 rpm for 24 h. Bacterial growth was monitored by measuring the OD_{600} at indicated time points. The experiments were repeated three times, and error bars indicate the standard deviation.

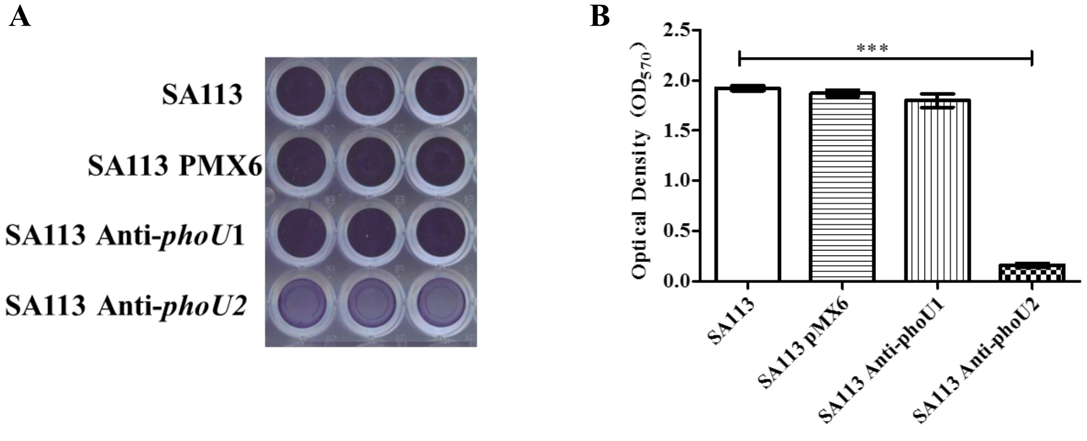

Supplementary Figure 3. Impacts of *phoU1* or *phoU2* silencing on the biofilm formation of SA113. Overnight cultures (12 h) of the indicated strains were diluted 1:200 with fresh TSB containing 125 ng/ml anhydrotetracycline (ATc) and incubated statically in 96-well polystyrene plates at 37°C for 6 h. The biofilms were washed with PBS and stained with crystal violet. The OD_{570} of the plates were measured. **(A)** Biofilm formation of the SA113, SA113 pMX6, SA113 Anti-*phoU1* and SA113 Anti-*phoU2* strains in the wells of 96-well polystyrene plates. **(B)** Semi-quantitative analysis of biofilm formation. The experiments were repeated three times, and error bars indicate the standard deviation. Significance differences between SA113 Anti-*phoU2* and SA113 were indicated by ***, P<0.001.

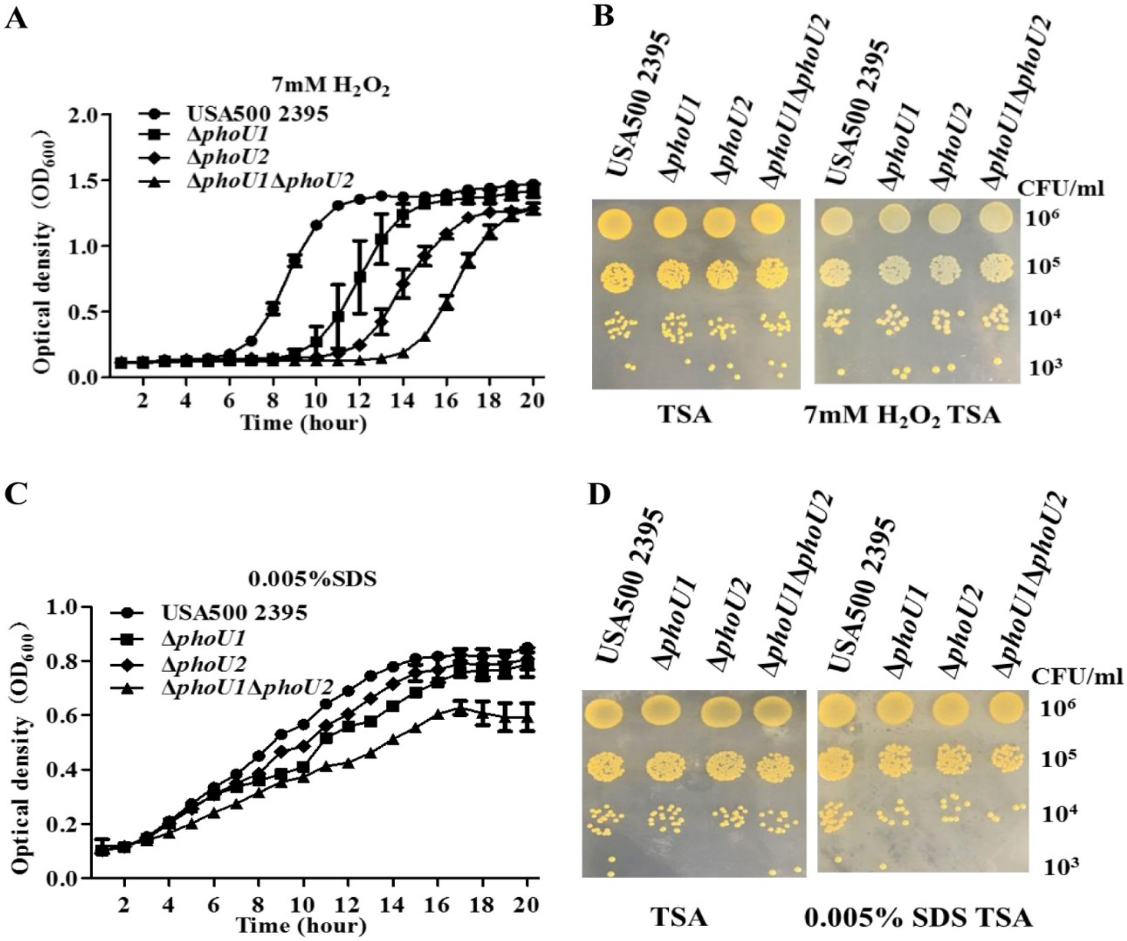

Supplementary Figure 4. Deletion of *phoU1* or *phoU2* increased the sensitivity to H_2O_2 and SDS of *S. aureus*. Overnight cultures (12 h) of the Δ*phoU1*, Δ*phoU2*, Δ*phoU1*Δ*phoU2* and USA500 2395 strains were diluted 1:200 into TSB containing either **(A)** hydrogen peroxide (H_2O_2; 7 mM) or **(C)** sodium dodecyl sulfate (SDS; 0.005%), then grown at 37°C with shaking at 220 rpm and monitored by measuring the OD_{600} at indicated time points, until 20 h. Overnight cultures (12 h) of the Δ*phoU1*, Δ*phoU2*, Δ*phoU1*Δ*phoU2* and USA500 2395 strains were 10-fold serial diluted and spotted 5 µl onto TSB agar containing either **(B)** H_2O_2 (7 mM) or **(D)** SDS (0.005%), then were incubated overnight at 37°C. The experiments were repeated three times, and error bars indicate the standard deviation.

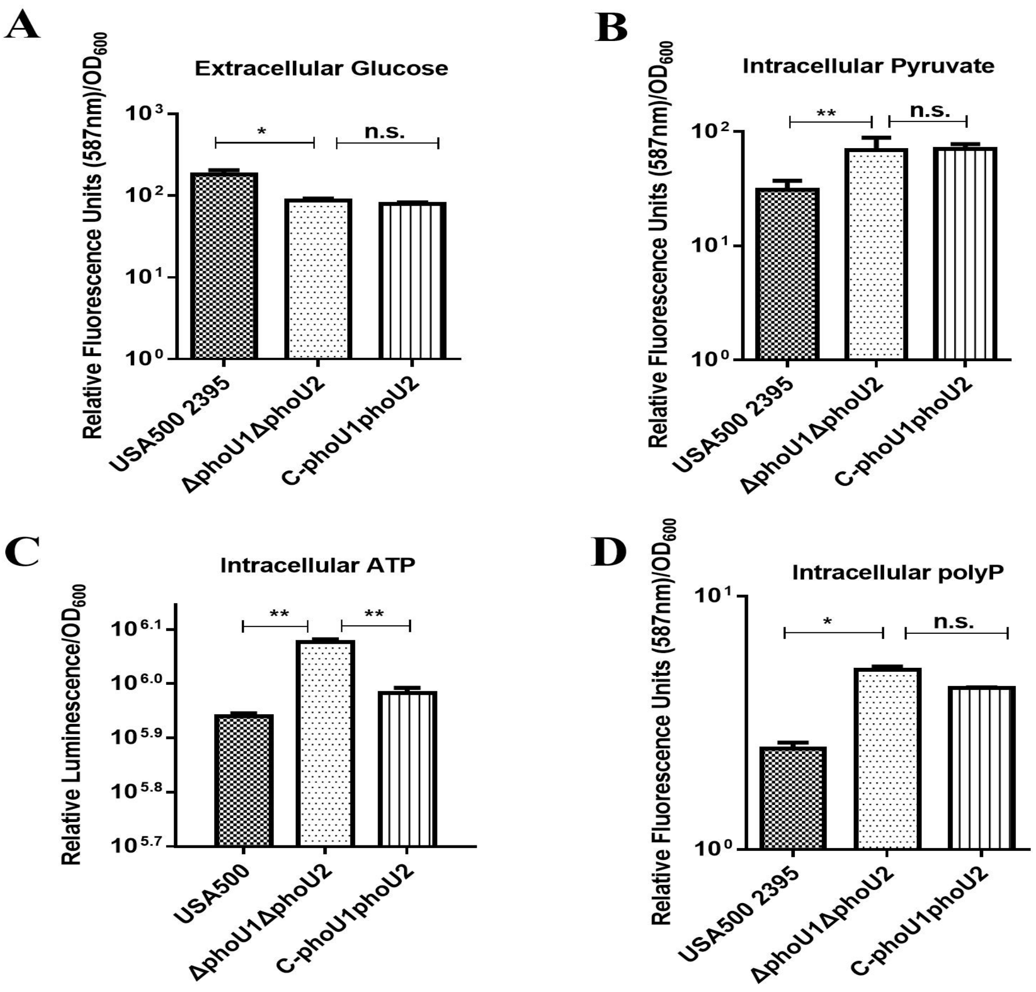

Supplementary Figure 5. Extracellular glucose, intracellular pyruvate, ATP and Intracellular polyP levels in USA500 2395, Δ*phoU1*Δ*phoU2* and C-Δ*phoU1*Δ*phoU2*of *S. aureus*. (A) Extracellular glucose. Bacteria were grown for 12 h, then the culture supernatant was harvested. Fluorescence signal was determined at an excitation of 535 nm and an emission of 587 nm. **(B)** Intracellular pyruvate. Bacteria were grown for 12 h, then the cells were homogenized by OD_{600} and lysed with 0.1 mm glass-silica beads in a BeadBeater apparatus. Centrifugation to get the supernatant. The fluorescence signal was determined at an excitation of 535 nm and an emission of 587 nm. **(C)** Intracellular ATP. Bacteria were grown for 12 h, then the cells were homogenized by OD_{600} and added equal to the volume of BacTiter-Glo™ Reagent. **(D)** Data are the means±SDs from three independent experiments. Luminescence was determined. Intracellular polyP. Bacteria were grown 12 h, then the cells were homogenized by OD_{600} and incubated 15mins with DAPI. The fluorescence signal was determined at an excitation of 415 nm and an emission of 550 nm. The experiments were repeated three times, and error bars indicate the standard deviation. Significance differences between Δ*phoU1*Δ*phoU2* and USA500 2395 were indicated by **, $P<0.01$; *, $P<0.05$; n.s., no significance.

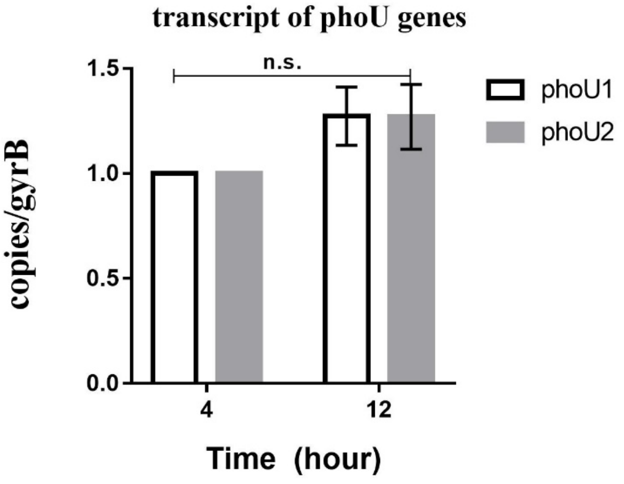

Supplementary Figure 6. PhoU genes transcript in the *S. aureus* USA500 2395. RNA was isolated from *S. aureus* USA500 2395 when grown to 4 h and 12 h in TSB medium. The expression of select transcripts was measured using quantitative reverse transcription-PCR, and the results normalized to the *gyrB* transcript level. The experiments were repeated three times, and error bars indicate the standard deviation. n.s., no significance.

Supplementary Tables

Supplementary Table 1. Plasmids and bacterial strains used in the present study

Plasmids and bacterial strains	Description	Source or reference
**Bacterial strains**		
S. aureus USA500	MRSA strain from a clinical sample	Diep et al., 2006
S. aureus USA300_FPR3757	MRSA strain from a clinical sample	Diep et al, 2006
S aureus SA113	an MSSA strain, derivate from NCTC8325	Iordanescu et al., 1976
USA500 Δ*phoU1* mutant	*phoU1* deletion mutant using USA500 as parental strain	This study
USA500 Δ*phoU2* mutant	*phoU2* deletion mutant using USA500 as parental strain	This study
USA500 Δ*phoU1* Δ*phoU2* mutant	*phoU1 phoU2* deletion mutant using USA500 as parental strain	This study
Δ*phoU1/pCN51::phoU*	Δ*phoU1* mutant complemented with plasmid pCN51 harboring *phoU2* gene	This study
Δ*phoU/pCN51*	Δ*phoU1* mutant complemented with plasmid pCN51	This study

Δ*phoU2/pCN51::phoU2*	Δ*phoU2* mutant complemented with plasmid pCN51 harboring *phoU2* gene	This study
Δ*phoU2/pCN51*	Δ*phoU2* mutant complemented with plasmid pCN51	This study
DC10B	Δ*dcm* in the *DH10B* background; Dam methylation only	Monk et al, 2006
**Plasmids**		
pKOR1	temperature-sensitive *E.coli*(Amp^r)-*Staphylococcus* shuttle(Cm^r) vector	Renji hospital, prof Li Min
pKOR1-Δ*phoU1*	recombinant plasmid	This study
pKOR1-Δ*phoU2*	recombinant plasmid	This study
pMX6	a ATc-inducible asRNA expression vector, Erm^r	Tao Xu et al, 2017
pMX6-*phoU2* asRNA	a ATc-inducible asRNA expression vector, Erm^r the *phoU2* asRNA was cloned into plasmid pMX6	This study
pCN51	a Cd^{2+} inducible shuttle plasmid, Erm^r	Wageningen University, Holland
pCN51- *phoU1*	a Cd^{2+} inducible shuttle plasmid, Erm^r ,the *phoU1* gene with its own promoter was cloned into plasmid pCN51	This study
pCN51- *phoU2*	a Cd^{2+} inducible shuttle plasmid, Erm^r ,the *phoU2* gene its own promoter was cloned into plasmid pCN51	This study
pRB473	shuttle plasmid(Cm^r)	Department of Microbial Genetics, Tübingen, University
pRB473- *phoU1*	shuttle plasmid(Cm^r), *phoU1* gene with its own promoter was cloned into plasmid pRB473	This study

Supplementary Table 2. Effect of *phoU1* or *phoU2* deletion on antibiotic susceptibility of *S. aureus*

Antibiotic	MIC/MBC (μg/ml) for indicated strain		
	USA500 2395	Δ*phoU1*	Δ*phoU2*
Vancomycin	0.5/0.5	0.5/0.5	0.5/0.5
Levofloxacin	0.25/0.5	0.25/0.5	0.25/0.5

Gentamicin	0.5/1	0.5/1	0.5/1
[a]Daptomycin	1/2	1/2	1/2

Supplementary Table 3. Primers used for construction of *phoU1* and *phoU2* deletion, complement or asRNA silencing mutants

Primer	Primer sequence（5'- 3'）	Underlined
phoU1-up-s	GGGGACAAGTTTGTACAAAAAAGCAGGCTCGGTGCGTTAAGTGTTTTAG	attB1
phoU1-up-as	CGGggtaccCTTAAAGTTATTACTATAAAATCC	kpnI
phoU1-down-as	GGGGACCACTTTGTACAAGAAAGCTGGGTGTTGCATCGGCAGGCATTA	attB2
phoU1-down-s	CGGggtaccTATATATCAACCAAACCTTCCT	kpnI
phoU2-up-s	GGGGACAAGTTTGTACAAAAAAGCAGGCTGATGAGTTAGAGAATTATAGTAT	attB1
phoU2-up-as	CGGggtaccTTAAAATCCTCCATTTAAGCGA	kpnI
phoU2-down-as	GGGGACCACTTTGTACAAGAAAGCTGGGTTAAACCTGCTAACAATGCTGA	attB2
phoU2-down-s	CGGggtaccGGGGAGTATATATTTATGTCAT	kpnI
phoU1-s	TTATTGTTCGTAATGTGTACCT	
phoU1-as	ATGGCAATAATTAGACAACGAT	
phoU2-s	TGGTTCAATTAGAAGAGATGG	
phoU2-as	TGCTACGATTTGACATTTATCA	
phoU1-promoter-s	aggtcgactctagaggatccAGGGATTTATGTCCCAGCCATT	pRB473 overlapping sequence
phoU1-promoter-as	tctaattattgccatTGAAATATCCTCCCTGTATGAA	pRB473-*phoU1*-s overlapping sequence
pRB473-*phoU1*-s	agggaggatatttcaATGGCAATAATTAGACAACGAT	*phoU1*-promoter-as overlapping sequence
pRB473-*phoU1*-as	attcgagctcggtaccTTATTGTTCGTAATGTGTACCT	pRB473 overlapping sequence
pCN51-*phoU2*-s	atgtataattttgatatcAAGTTAAGTTTCGGAATTGTTA	pCN51 overlapping sequence
pCN51-*phoU2*-as	aaatcagtttttgatatcATGACATAAATATATACTCCCC	pCN51 overlapping sequence
**As- *phoU1*s**	gaagatctTCGCTTAAATGGAGGATTTTAA	Bgl II
**As- *phoU1*as**	agcggccgAAATCTAAATGTGTATTGAAATC	EagI

**As-*phoU2*s**	gaagatctTATATAATGGCAATAATTAGACAAC	Bgl II
**As- *phoU2*as**	agcggccgCAATACTCACATAGACATTTG	EagI

Supplementary Table 4. qRT-PCR primers for detection of *phoU1* and *phoU2* transcriptional level in USA500 2395 mutations

**Primers**	**Sequences（5'-3'）**
gyrB-F	ACATTACAGCAGCGTATTAG
gyrB-R	CTCATAGTGATAGGAGTCTTCT
phoU1-F	CAGCGATGTTAATAATATGAT
phoU1-R	ATAATTGAGCGTGATGAA
phoU2-F	ATCGTGCTGCTATTGAAT
phoU2-R	ATTGGTGTGATAAATGTTTGAT

Supplementary Table 5 Virulence-related differentially expressed genes of Δ*phoU1* or Δ*phoU2* compared to USA500 2395

Functional classification	DEG ID	DEG Name	Description	Δ*phoU1* Ratio of expression		Δ*phoU2* Ratio of expression		Important biological function	References
				RNA-seq	RT-qPCR	RNA-seq	RT-qPCR		
Secretion protein	CH51_RS01375	*esxA*	virulence factor EsxA	ND	ND	0.22	ND	T7SS is composed of four membrane-associated proteins (EsaA, EssA, EssB, and EssC), three soluble cytosolic proteins (EsaB, EsaE, and EsaG), and five secreted virulence factors (EsxA, EsxC, EsxB, EsxD, and EsaD). T7SS is a dedicated virulence protein-secretion pathway that enables long-term survival of the bacteria in abscesses, where they are protected from host immune cells.	Michael S. Lopez, 2017, PNAS
	CH51_RS01380	*esaA*	type VII secretion protein EsaA	0.37	0.35±0.31	0.31	0.65±0.27		
	CH51_RS01385	*essA*	protein EssA	0.18	0.03±0.01	0.28	0.03±0.01		
	CH51_RS01395	*essB*	protein EssB	0.39	ND	0.35	ND		
	CH51_RS01400	*essC*	protein EssC	0.46	ND	0.36	ND		
Extracellular enzyme	CH51_RS10905	*scpA*	staphopain A	ND	ND	0.34	ND	Staphopain A/B is a staphylococcal cysteine protease and staphostatin A/B is its specific inhibitor.	Rzychon M, 2003, Mol Microbiol.
	CH51_RS10910	*sspB*	staphostatin A	0.3	0.02±0.01	0.43	0.28±0.03		
	CH51_RS05325	*scpB*	staphostatin B	0.3	ND	0.36	ND	The Ssp glutamyl endopeptidase moderates adhesion of S. aureus to fibronectin by degrading cell surface fibronectin-binding proteins.	Kelly Rice, 2011, Infect. Immun.
	CH51_RS05330	*sspC*	staphopain B	0.32	0.29±0.09	0.41	0.7±0.31		
	CH51_RS05335	*sspA*(V8)	serine protease	0.26	0.45±0.16	0.4	0.75±0.38		

	CH51_RS10165	*splF*	serine protease	0.14	0.42±0.05	0.08	ND	The Spl serine proteases modulate Staphylococcus aureus protein production and virulence in a rabbit model of pneumonia. SplB and SplC were shown to degrade casein	Paharik AE, 2016, mSphere
	CH51_RS10170	*splE*	serine protease	0.16	0.42±0.05	0.08	1.02±0.45		
	CH51_RS10175	*splD*	serine protease	0.11	ND	0.1	ND		
	CH51_RS10180	*splC*	serine protease	0.14	ND	0.09	ND		
	CH51_RS10185	*splB*	serine protease	0.13	ND	0.08	ND		
	CH51_RS10190	*splA*	serine protease	0.18	1.09±0.31	0.08	0.08±0.01		
Cytotoxin	CH51_RS11465	*lukG*	leukocidin LukGH subunit	0.18	0.09±0.02	0.3	0.22±0.03	LukGH have potent cytolytic activity in vitro, and toxins are proinflammatory in vivo.	Yanai M, 2014, PLoS One.
	CH51_RS11470	*lukH*	leukocidin LukGH subunit	0.25	0.17±0.06	0.3	0.14±0.03		
	CH51_RS10250	*lukD*	leukocidin LukED	0.23	0.17±0.14	0.33	ND		
	CH51_RS13695	*hlgA*	gamma-hemolysin HlgAB subunit	0.27	0.19±0.03	0.45	0.09±0.01	γ-hemolysin has a broad range of target cells, including erythrocytes, PMNs, monocytes and lymphocytes.	András N. Spaan, 2015, Nat Commun
	CH51_RS13700	*hlgC*	gamma-hemolysin HlgCB subunit	0.3	0.19±0.14	0.36	0.21±0.14		
	CH51_RS13705	*hlgB*	gamma-hemolysin HlgAB/HlgCB subunit	0.45	0.77±0.24	0.42	0.42±0.24		
	CH51_RS06245	*hla*	alpha-hemolysin	0.27	0.09±0.01	0.45	0.83±0.31	Hla has been recognized as an important cause of injury in the context of both skin necrosis and lethal infection.	Berube BJ, 2013, Toxins

	CH51_RS11020	*pmtD*	phenol-soluble modulin export ABC transporter permease subunit	0.43	ND	ND	ND	The transporter had a strong effect on virulence phenotypes, which is essential for bacterial growth.	Som S Chatterjee, 2013, Nature Medicine
	CH51_RS11030	*pmtB*	phenol-soluble modulin export ABC transporter permease subunit	0.47	ND	ND	ND		
Adhesive factor	CH51_RS06210	*fnp*	fibrinogen-binding protein	0.34	ND	ND	ND	Fibronectin-binding protein acts as Staphylococcus aureus invasin via fibronectin bridging to integrin α-5β1	Bhanu Sinha, 1999, Cellular Microbiology
	CH51_RS06215	*fnp*	fibrinogen-binding protein	0.28	ND	ND	ND		
	CH51_RS14165	*fnpA*	fibronectin-binding protein A	2.06	1.35±0.31	ND	ND		
Others	CH51_RS11100	*sak*	staphylokinase	0.47	0.18±0.03	0.27	0.8±0.13	Staphylokinase is a plasminogen activator protein that is secreted by many Staphylococcus aureus strains. Sak also offers protection by binding and inhibiting specific antimicrobial peptides.	Leonard Nguyen, 2016, Scientific Reports
	CH51_RS13685	*sbi*	immunoglobulin-binding protein	0.46	0.46±0.16	0.33	0.72±0.24	Sbi acts as a complement inhibitor.	Katrin H, 2013, PNAS
	CH51_RS00585	*SasD*	cell-wall-anchored protein	0.3	0.21±0.04	0.43	0.13±0.02		
	CH51_RS03160	*VraX*	protein VraX	0.43	ND	0.22	ND	Inhibited the classical pathway of the complement system	Yan J, 2017, Mol Immunol
Regulator factor	CH51_RS01020	*hptR*	DNA-binding response regulator	ND	ND	0.47	ND	Mutational studies revealed that disruption of the hptA gene impaired the growth of bacteria	Park JY,2015,Infect Immun.

	CH51_RS03625	*graR*	DNA-binding response regulator	2.51	ND	ND	ND	The GraS/GraR two-component system has been shown to control cationic antimicrobial peptide (CAMP) resistance in the major human pathogen *Staphylococcus aureus*.	Falord M, 2011, PLoS One.
	CH51_RS03630	*graS*	sensor histidine kinase	2.76	ND	ND	ND		
	CH51_RS03870	*saeS*	two-component sensor histidine kinase	0.48	ND	ND	ND	*saeRS* activates the transcription of *fnbA*, *coa*, and *hla*.	Steinhuber A,2003,J Bacteriol
	CH51_RS09485	*phoR*	sensor histidine kinase	ND	ND	3.64	ND	Unknown	No reported
	CH51_RS09490	*phoP*	DNA-binding response regulator	ND	ND	3.42	ND		
	CH51_RS03415	*sarA*	transcriptional regulator	0.35	0.69±0.33	0.35	ND	Regulated fibronectin binding proteins (fnbA and fnbB), hemolysins (hla, hld, hlgB and hlgC), and serine proteases (splA splB, splD and splF).	P. M. Dunman, 2001, J Bacteriol.
	CH51_RS13015	*sarR*	HTH-type transcriptional regulator	ND	ND	0.37	ND	SarR on binding to the sarpromoter is a reduction in P1 and in the combined (P2-P3-P1)sar promoter activity, thus leading to a decrease in SarA protein expression.	Adhar Manna,2001,Infect Immun.
	CH51_RS13505	*sarZ*	transcriptional regulator	ND	ND	0.46	ND	SarZ promotes the expression of virulence factors and represses biofilm formation by modulating SarA and agr.	Sandeep Tamber, 2009,Infect Immun.
	CH51_RS06740	*codY*	GTP-sensing pleiotropic transcriptional regulator	2.38	2.59±0.41	0.49	ND	CodY is a repression of the agr system.	Majerczyk CD, 2010, J Bacteriol.

	CH51_RS09890	*rot*	MarR family transcriptional regulator	ND	ND	0.44	0.16±0.03	Rot negatively regulates hlb, hlg gene.	B. Saïd-Salim,2003, J Bacteriol

[a] ND, not done.
